# Review of the Genus *Sinacroneuria* (Plecoptera: Perlidae) from China, with Descriptions of Five New Species †

**DOI:** 10.3390/insects17080826

**Published:** 2026-08-10

**Authors:** Yingying Wang, Ruijun Liu, Guoquan Wang, Keda Chen, Weihai Li

**Affiliations:** 1 Guangxi Key Laboratory of Agric-Environment and Agric-Products Safety and National Demonstration Center for Experimental Plant Science Education, Agricultural College, Guangxi University, Nanning 530004, China; wyy5657@163.com; 2 Henan International Joint Laboratory of Taxonomy and Systematic Evolution of Insecta, Henan Institute of Science and Technology, Xinxiang 453003, China; histlruijun@163.com; 3 Guangxi Dayaoshan National Nature Reserve Management Center, Laibin 545703, China; chenkedagood@126.com; 4 Observation and Research Station on Water Ecosystem in Danjiangkou Reservoir of Henan Province, Nanyang 474450, China

**Keywords:** stonefly, *Sinacroneuria*, review, new species, taxonomy, China

## Abstract

The stonefly genus *Sinacroneuria* Yang and Yang, 1995 (Plecoptera: Perlidae) is restricted to East Asia and represents an important component of freshwater biodiversity. However, the taxonomy of Chinese *Sinacroneuria* has not been comprehensively revised, resulting in difficulties in species identification. In this study, we conducted a systematic review of the Chinese species based on morphological characters. Before the present study, *Sinacroneuria* comprised 20 described species worldwide, 18 of which were recorded from China. The five new species bring these totals to 25 species worldwide and 23 in China, representing increases of 25 percent and 27.8 percent, respectively. All Chinese species are diagnosed, and five new species, *S. dayaoshan* sp. nov., *S. fengyanghu* sp. nov., *S. magnimaculata* sp. nov., *S. similiwui* sp. nov., and *S. yunjishan* sp. nov., are described. An updated identification key to all Chinese species is also provided. These results improve our understanding of the diversity and taxonomy of *Sinacroneuria* and provide an important taxonomic reference for future biodiversity, ecological, and biomonitoring studies.

## Simple Summary

The perlid genus *Sinacroneuria* Yang and Yang, 1995 (Plecoptera: Perlidae) is restricted to East Asia and includes species that are sensitive to environmental changes, making them useful indicators of freshwater ecosystem conditions. However, the species diversity and identification of Chinese *Sinacroneuria* remain poorly understood. Before this study, 18 species of *Sinacroneuria* were known from China. Here, we conducted a comprehensive morphological review of Chinese species and describe five new species, as well as the females of three previously known species for the first time. The number of Chinese *Sinacroneuria* species is therefore increased to 23, and an updated identification key is provided for all known species. This study improves our understanding of the diversity of *Sinacroneuria* and provides essential information for future biodiversity and ecological studies.

## 1. Introduction

The genus *Sinacroneuria* Yang and Yang, 1995 belongs to the subfamily Acroneuriinae and is restricted to East Asia [1,2]. It was established as a monotypic genus for *S. orientalis* by Yang and Yang [3]. Subsequent taxonomic studies transferred four species originally described under *Acroneuria* to *Sinacroneuria*. *Sinacroneuria yiui* was originally described in *Mesoperla* and subsequently transferred to *Acroneuria* before being assigned to *Sinacroneuria*. These studies further expanded the genus through the description of numerous new species, systematic revisions, and the publication of identification keys [2,4,5,6]. Before the present study, *Sinacroneuria* comprised 20 extant species, including 18 from China and one species each from Vietnam and Japan [1,7,8].

Despite the considerable progress made in recent decades, the taxonomy of *Sinacroneuria* remains incomplete. Female genitalia have been described for only a limited number of species, restricting comparative studies and reliable species identification [6,7,9]. Furthermore, continued taxonomic investigations continue to reveal previously undocumented diversity within the genus.

In the present study, we review the Chinese species of *Sinacroneuria* and update their diagnoses based on newly examined material. Five new species, *S. dayaoshan* Y.Y. Wang, G.Q. Wang and W.H. Li sp. nov., *S. fengyanghu* Y.Y. Wang, G.Q. Wang and W.H. Li sp. nov., *S. magnimaculata* Y.Y. Wang, G.Q. Wang and W.H. Li sp. nov., *S. similiwui* Y.Y. Wang, G.Q. Wang and W.H. Li sp. nov., and *S. yunjishan* Y.Y. Wang, G.Q. Wang and W.H. Li sp. nov., are described from Anhui, Guangdong, Guangxi, and Zhejiang, China. In addition, the female genitalia of three previously described species are documented for the first time, and an updated identification key to the Chinese species of *Sinacroneuria* is provided.

## 2. Materials and Methods

### 2.1. Material Depository

The holotypes and paratypes of the new species are deposited in the following collections:

HIST—Insect Collection of Henan Institute of Science and Technology, Xinxiang, China.

NHMC—the Natural History Museum of China, Beijing, China.

Type specimens of previously described species of the genus *Sinacroneuria* are deposited in the following collections:

CAUC—Entomological Museum of China Agricultural University, Beijing, China.

HNHM—Hungarian Natural History Museum, Budapest, Hungary.

ICYZU—the Insect Collection of Yangzhou University.

USNM—the United States National Museum of Natural History, Washington.

ZFMK—the Zoologisches Forschungsmuseum Alexander Koenig, Bonn.

### 2.2. Morphological Study

Specimens used in this study were collected by hand, light trap, or Malaise trap. Historical specimens were stored in 75% ethanol, whereas recently collected material was preserved in 95% ethanol. Male terminalia were removed from the abdomen and soaked in 10% NaOH. Specimens were examined with the aid of a Nexcope NSZ818 dissecting microscope. Color photographs were taken using an Imaging Source CCD camera attached to a Leica M205 FCA stereomicroscope. Photos were enhanced using Adobe Photoshop 2025. The morphological terminology follows [2,6].

Abbreviations for morphological terms used in key and figures

ls = lateral sclerite

ms = median sclerite

pp = paraproct

sgp = subgenital plate

T9 = tergum 9

T10 = tergum 10

## 3. Results

### 3.1. Taxonomic Account of the Genus Sinacroneuria

*Nishineuria* Uchida, 1990: 127, nomen nudum; proposed for the manuscript name *N. cornuta*, subsequently validated as *Sinacroneuria acuticornis* Uchida in [7]; see ICZN Article 11.

*Sinacroneuria* Yang & Yang, 1995: 1 [3]. Type-species: *Sinacroneuria orientalis*, by monotypy.

**Diagnosis.** Medium to large, macropterous species with a strongly sclerotized hammer and a well-developed Y-shaped aedeagal structure. Male terga 9–10 typically bear median patches of sensilla basiconica, and paraprocts are broad basally, heavily reflexed inward and forward. The genus *Sinacroneuria* is distinguished from *Acroneuria* by the well-developed Y-shaped aedeagal structure.

**Distribution.** Oriental (23 species), and Palaearctic (2 species) [1].

### 3.2. Synoptic List of Chinese Species of Sinacroneuria

This study documents the following 23 species in China, including 5 new species, as listed below.

*S. aequalis* Liu, Cao & Li, 2025

*S. bicornuata* Stark & Sivec, 2008

*S. complana* Xiang & Du, 2022

*S. dabieshana* Li & Murányi, 2014

*S. dayaoshan* sp. nov.

*S. fengyanghu* sp. nov.

*S. fujianensis* Sivec & Stark, 2020

*S. huzhengkuni* Du & Huo, 2020

*S. lateralis* Sivec & Stark, 2020

*S. lii* Huo & Du, 2020

*S. longiprojecta* Du & Huo, 2022

*S. longwangshana* (Yang & Yang, 1998)

*S. magnimaculata* sp. nov.

*S. obscura* Li & Murányi, 2017

*S. orientalis* Yang & Yang, 1995

*S. parallela* Xiang & Du, 2022

*S. quadriplagiata* (Wu, 1938)

*S. similiwui* sp. nov.

*S. sinica* (Yang & Yang, 1998)

*S. transversa* Liu, Cao & Li, 2025

*S. wui* (Yang & Yang, 1998)

*S. yiui* (Wu, 1935)

*S. yunjishan* sp. nov.

### 3.3. Taxonomic Accounts of Chinese Species of Sinacroneuria

***Sinacroneuria aequalis* Liu, Cao & Li, 2025**

**Figure 1:**
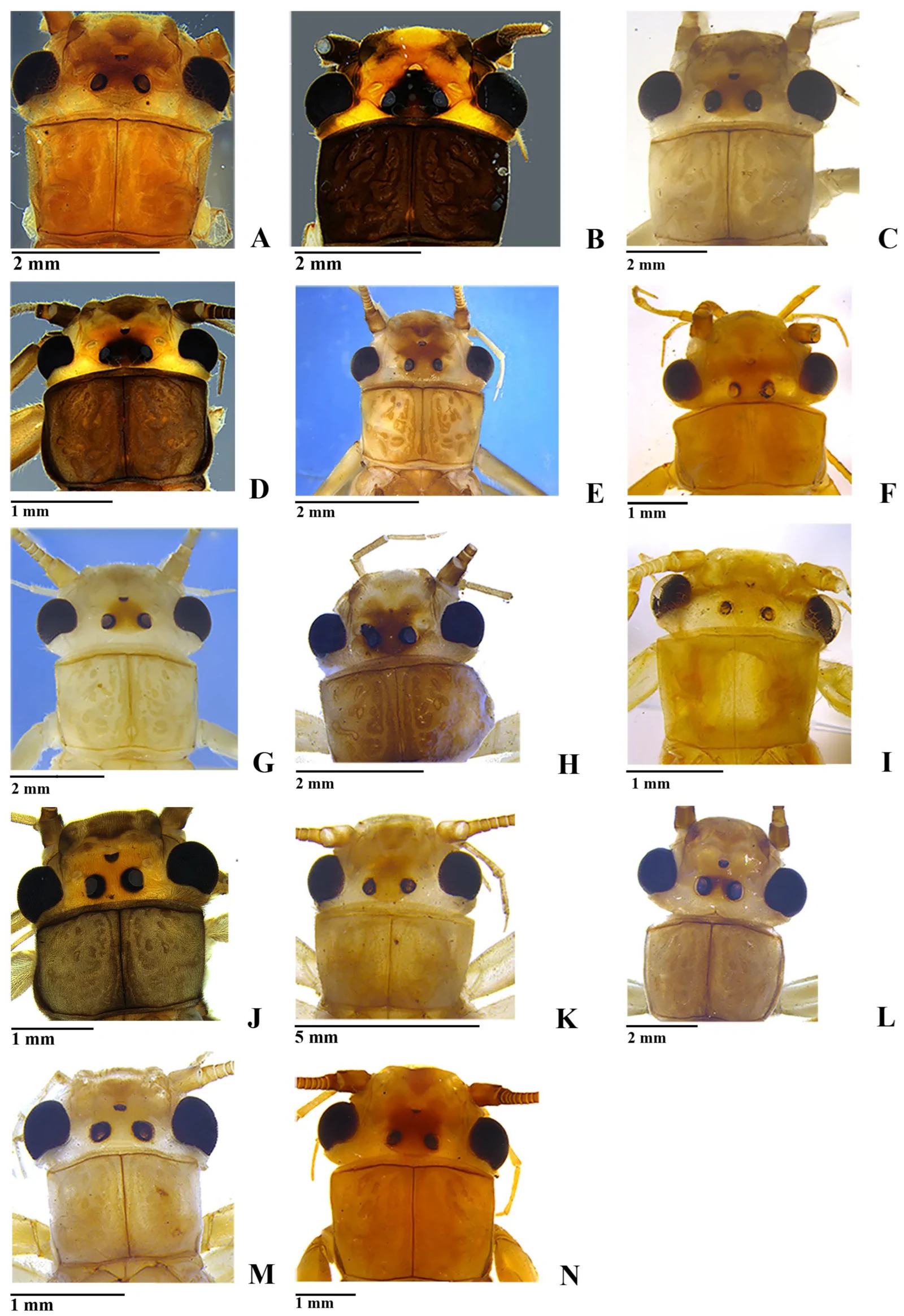
Head and pronotum of the genus *Sinacroneuria*, male. (**A**) *S. aequalis*; (**B**) *S. bicornuata*; (**C**) *S. dabieshana*; (**D**) *S. huzhengkuni*; (**E**) *S. lii*; (**F**) *S. longiprojecta*; (**G**) *S. longwangshana*; (**H**) *S. obscura*; (**I**) *S. orientalis*; (**J**) *S. parallela*; (**K**) *S. quadriplagiata*; (**L**) *S. sinica*; (**M**) *S. transversa*; (**N**) *S. wui.*

**Figure 2:**
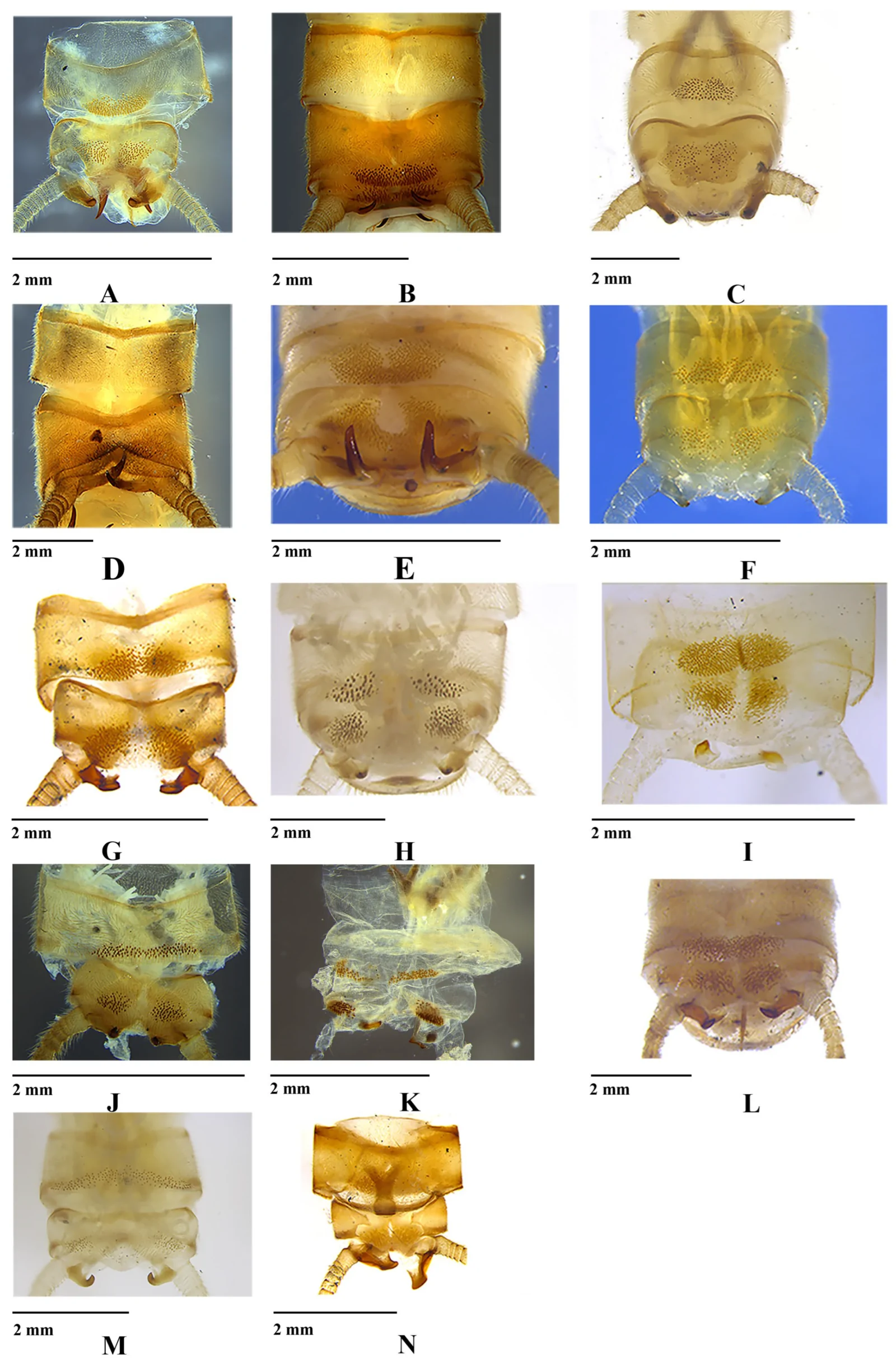
Genitalia of the genus *Sinacroneuria*, male. (**A**) *S. aequalis*; (**B**) *S. bicornuata*; (**C**) *S. dabieshana*; (**D**) *S. huzhengkuni*, 2020; (**E**) *S. lii*; (**F**) *S. longiprojecta*; (**G**) *S. longwangshana*; (**H**) *S. obscura*; (**I**) *S. orientalis*; (**J**) *S. parallela*; (**K**) *S. quadriplagiata*; (**L**) *S. sinica*; (**M**) *S. transversa*; (**N**) *S. wui.*

**Figure 3:**
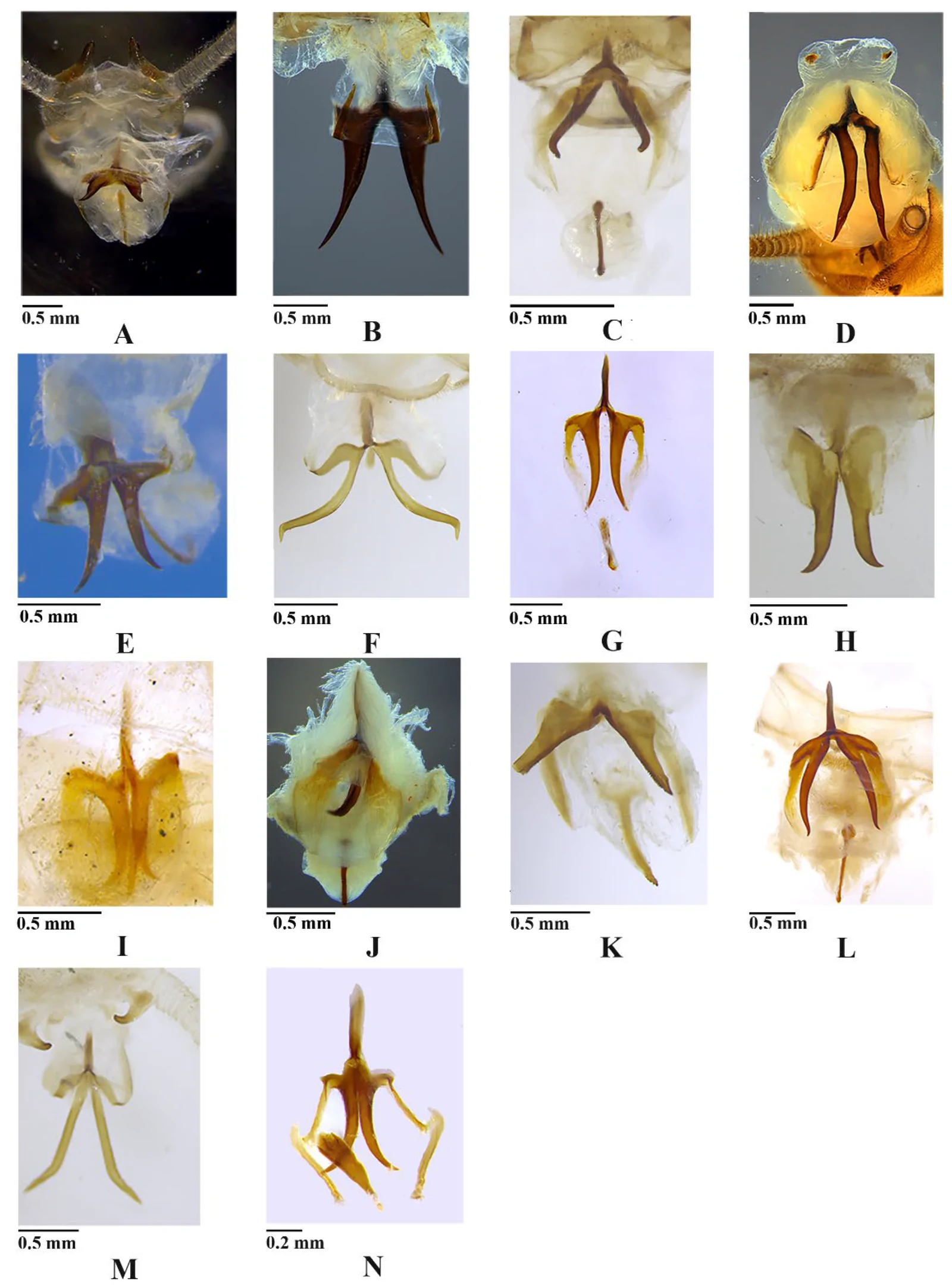
Aedeagus of the genus *Sinacroneuria*, male. (**A**) *S. aequalis*; (**B**) *S. bicornuata*; (**C**) *S. dabieshana*; (**D**) *S. huzhengkuni*, 2020; (**E**) *S. lii*; (**F**) *S. longiprojecta*; (**G**) *S. longwangshana*; (**H**) *S. obscura*; (**I**) *S. orientalis*; (**J**) *S. parallela*; (**K**) *S. quadriplagiata*; (**L**) *S. sinica*; (**M**) *S. transversa*; (**N**) *S. wui.*

Chinese common name: **等长华钮𧐐**

(Figure 1A, Figure 2A and Figure 3A)

*Sinacroneuria aequalis* Liu, Cao & Li, 2025: 83 [2]. HT: ♂, China, Fujian, Nanping City, Mt. Wuyi; HIST.

**Specimens examined.** Holotype. ♂, China, Fujian Province, Nanping City, Mt. Wuyi, 31-V-1990, 27°45′ N, 117°40′ E, 1150 m; Lianfang Yang and Changhai Sun leg. (HIST).

Diagnosis. *Sinacroneuria aequalis* is distinguished from congeners by the following characters: (1) triocellate; (2) Y-stem almost as long as the Y-arms; (3) Y-arms with apices curved outward; (4) median sclerite longer than Y-arms.

Distribution. China: Fujian (Nanping city) [2].

Remarks. This species was recently described from a single male collected in Fujian Province; a detailed description and illustrations were provided by Liu et al. [2].

***Sinacroneuria bicornuata* Stark & Sivec, 2008**

**Figure 4:**
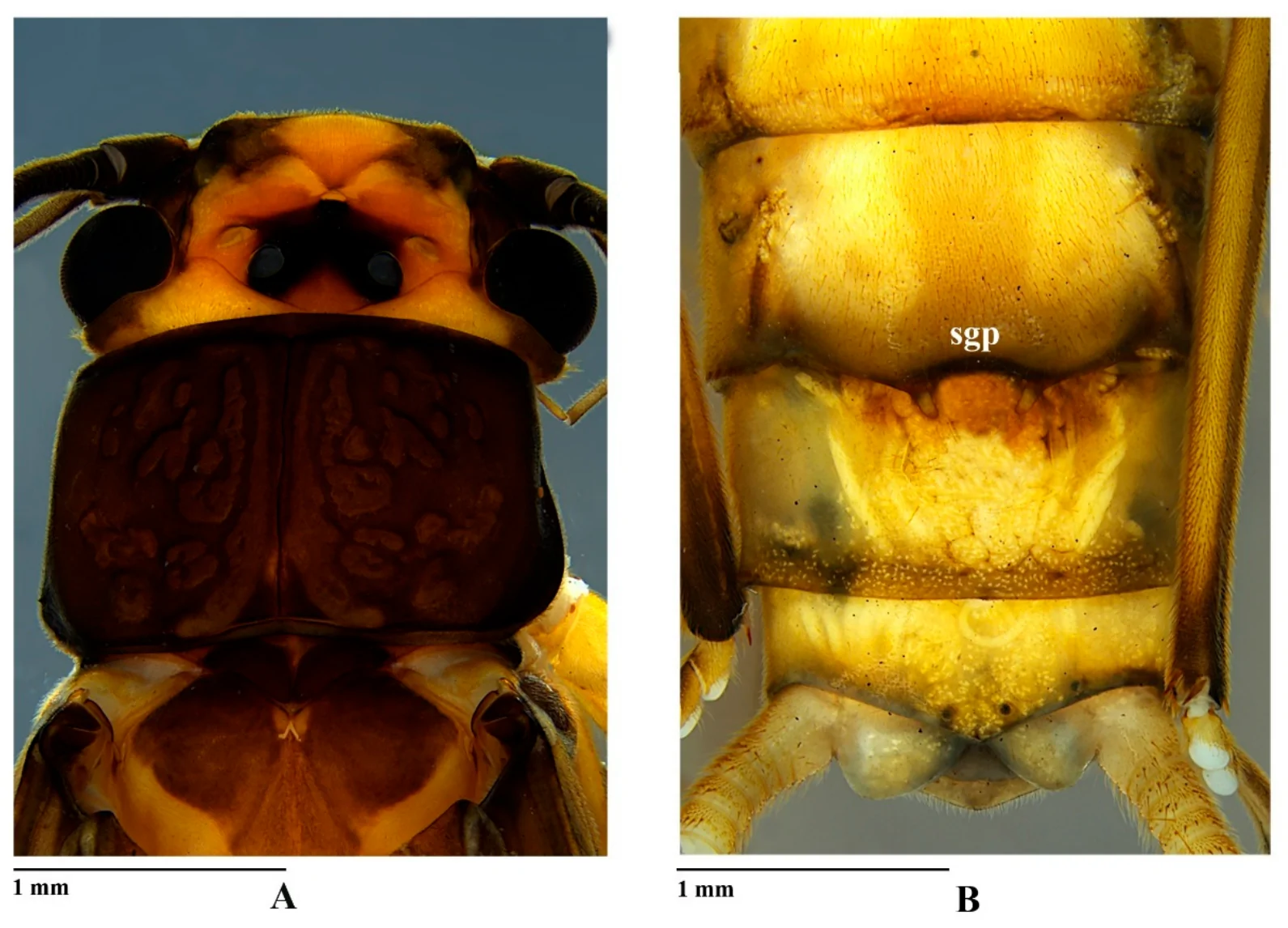
*Sinacroneuria bicornuata*, female. (**A**) Head and pronotum, dorsal view; (**B**) terminalia, ventral view.

Chinese common name: 双角华钮𧐐

(Figure 1B, Figure 2B, Figure 3B and Figure 4)

*Sinacroneuria bicornuata* Stark & Sivec, 2008: 152, 153 [8]. HT: ♂, China, Sichuan, Leshan City, Emeishan; USNM.

*Sinacroneuria bicornuata*: Li et al., 2014 [6]: 288; Murányi & Li, 2016 [9]: 184; Sivec & Stark, 2020: 102 [5].

**Specimens examined.** 3 ♂, China, Yunnan Province, Zhaotong City, Yiliang County, Xiaocaoba NNR, Jintan Town, 27°49′48″ N, 104°17′24″ E, 1766 m, 21-VII-2023, Light, Aijun Zhang leg. (NHMC); 2♂, China, Yunnan Province, Daguan County, Mugan Town, Sanjiangkou, 28°13′48″ N, 103°59′24″ E, 1800 m, 12-VII-2023, Light, Aijun Zhang leg. (NHMC); 1 ♀, same locality as above (Sanjiangkou), 15-VII-2023 (HIST).

Diagnosis. *Sinacroneuria bicornuata* is distinguished from congeners by the following characters: (1) triocellate; (2) Y-stem short, Y-arm broad basally and about 2.5 times as long as Y-stem; (3) Y-arms with apices curved outward; (4) median sclerite absent.

Distribution. China: Sichuan (Leshan City) [8], Yunnan (Zhaotong City).

Female (Figure 4). Forewing length ca. 23.4 mm. Sternum 8 mostly brown but median and posterior area with sclerotized bands; subgenital plate with a pair of widely spaced, finger-like lobes extending posteriorly to the anterior margin of sternum 9.

Remarks. Stark and Sivec [8] described *S. bicornuata* from Sichuan, China, with sketches and a detailed description. Li et al. [6] provided color photos in examining the holotype. In this study, we support a new provincial record in Yunnan Province based on male specimens and present the female specimens for the first time.

***Sinacroneuria complana* Xiang & Du, 2022**

Chinese common name: **扁平华钮𧐐**

*Sinacroneuria complana* Xiang & Du, 2022: 489, 490 [10]. HT: ♂, China, Zhejiang, Wenzhou City, Taishun County, Wuyanling National Nature Reserve; ICYZU.

Diagnosis. *Sinacroneuria complana* is distinguished from congeners by the following characters: (1) triocellate; (2) tergum 9 with several sensilla basiconica near the posterior margin; (3) Y-stem as long as Y-arm; (4) Y-arms with apices curved outward; (5) median sclerite with a thick base and a tapering apex bearing tiny spines.

Distribution. China: Zhejiang (Wenzhou City) [10].

Remarks. Specimens of *S. complana* were not examined in the present study; therefore, diagnosis was based on published descriptions and type photos. Xiang and Du [10] originally described this species from Zhejiang Province based on male specimens.

***Sinacroneuria dabieshana* Li & Murányi, 2014**

Chinese common name: **大别山华钮𧐐**

(Figure 1C, Figure 2C and Figure 3C)

*Sinacroneuria dabieshana* Li & Murányi, 2014: 285, 286, 287, 288 [6]. HT: ♂, China, Henan, Xinyang City, Xin County, Dabieshan; HIST.

**Specimens examined.** Holotype. ♂, China, Henan Province, Xinyang City, Xin County, Mt. Dabieshan, Liankangshan National Nature Reserve, 32°4′12″ N, 115°27′36″ E, light trap, 17-VI-2014, Weihai Li leg. (HIST). 8 males, Hubei Province, Huanggang City, Yingshan County, Mt. Dabieshan, Taohuachong, 30°58′59″ N, 116°2′29″ E, 694 m, light trap, 27-VI-2014, Mengchao Tan leg. (HIST).

Diagnosis. *Sinacroneuria dabieshana* is distinguished from congeners by the following characters: (1) triocellate, with the anterior ocellus smaller than the lateral ocelli; (2) sternum 9 with a large Y-shaped sclerite; (3) Y-arms about 4 times as long as the Y-stem; (4) Y-arms with apices curved inward; (5) median sclerite with an apical hook bearing tiny spines.

Distribution. China: Henan (Xinyang City), Hubei (Huanggang City) [6].

Remarks. This species was described in 2014 from male and female specimens collected in Henan and Hubei provinces.

***Sinacroneuria dayaoshan* Y.Y. Wang, G.Q. Wang & W.H. Li sp. nov.**

**Figure 5:**
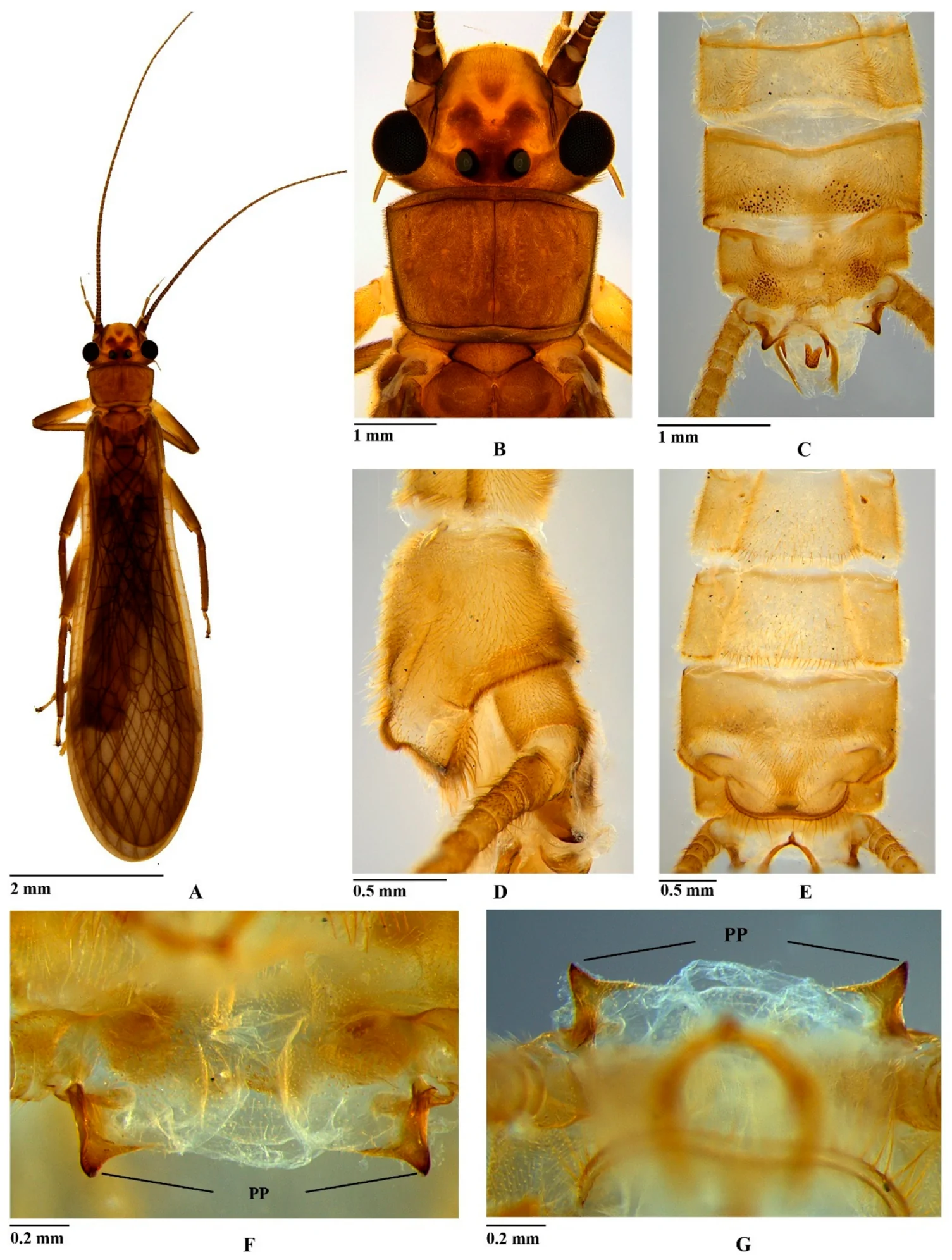
*Sinacroneuria dayaoshan* sp. nov., holotype, male, PLE037. (**A**) male habitus, dorsal view; (**B**) Head and pronotum, dorsal view; (**C**–**E**) terminalia: (**C**) dorsal view; (**D**) lateral view; (**E**) ventral view; (**F**) paraprocts, ventral view; (**G**) paraprocts, dorsal view.

**Figure 6:**
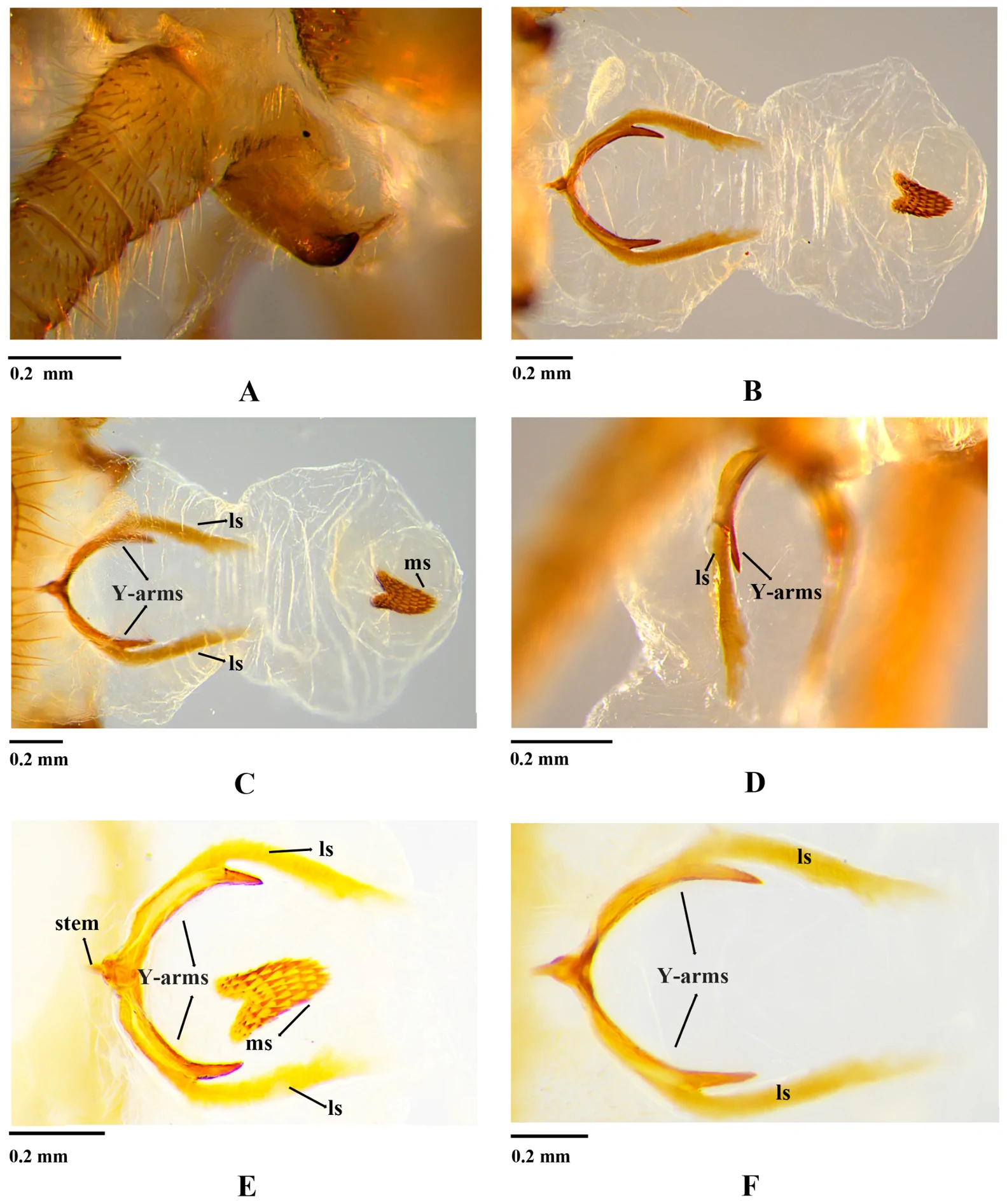
*Sinacroneuria dayaoshan* sp. nov., holotype, male, PLE037. (**A**) paraproct, dorsolateral view; (**B**–**D**) aedeagus: (**B**) dorsal view; (**C**) ventral view, (**D**). lateral view; (**E**) Y-shaped structure with median sclerite, dorsal view; (**F**) Y-shaped structure, dorsal view.

**Figure 7:**
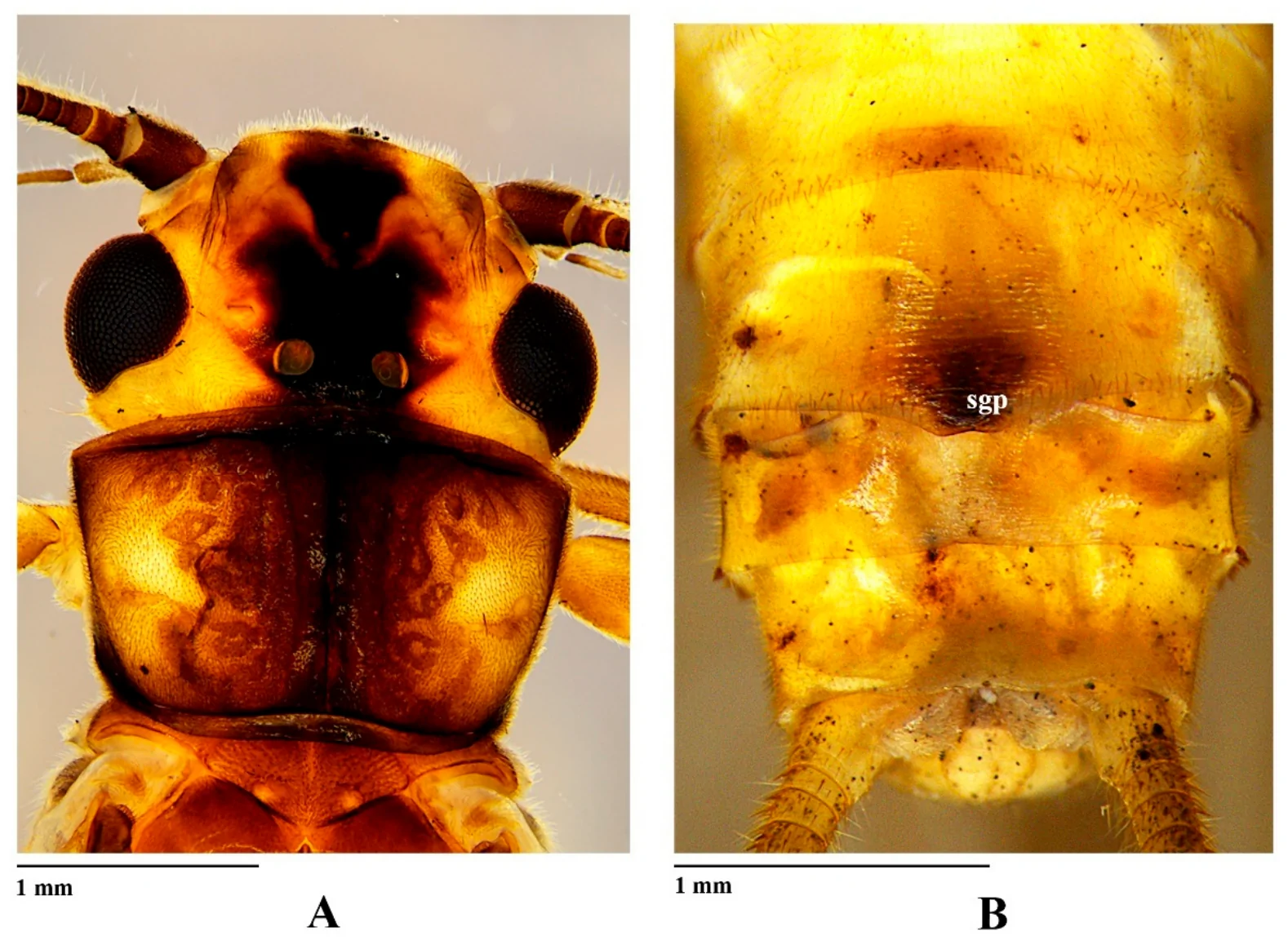
*Sinacroneuria dayaoshan* sp. nov., paratype, female. (**A**) Head and pronotum, dorsal view; (**B**) terminalia, ventral view.

Chinese common name: **大瑶山华钮𧐐**

(Figure 5, Figure 6 and Figure 7)

**Specimens examined. Holotype.** ♂, China, Guangxi Zhuang Autonomous Region, Laibin City, Jinxiu County, Dayaoshan National Nature Reserve, Yinshan Park, Light, 1172 m, 24°10′12″ N, 110°14′24″ E, 15-VII-2025, Xingnong Xu leg.; PLE037 (HIST); **Paratype**. 1♀, same data as holotype (NHMC); 1♂, China, Guangxi Zhuang Autonomous Region, Laibin City, Jinxiu County, Dayaoshan National Nature Reserve, 27-VII-2026, Shengtangshan, Light, 1162 m, 23°58′31″ N, 110°6′52″ E, Feng Lu leg. (NHMC)

**Diagnosis.** *Sinacroneuria dayaoshan* is distinguished from congeners by the following characters: (1) biocellate; (2) Y-arms about 4.5 times as long as the Y-stem; (3) Y-arms with apices curved inward; (4) heart-shaped median sclerite densely armed with strong triangular spines.

**Description.**

**Male**. Forewing length ca. 12.7 mm. General body color brown. Distance between ocelli as wide as the diameter of the ocellus. Head with a small brown spot on the anterior frons and a large dark brown spot covering ocellar triangle, extending laterally under pale M line; lappets dark brown (Figure 5A,B); antennae dark brown. Pronotum brown with rugosities (Figure 5A,B); wing membrane transparent and pale brown, with veins dark brown; legs brown. Abdominal segments brown.

**Male genitalia.** Tergum 9 with two sensilla patches posteriorly, each consisting of many larger triangular sensilla basiconica (Figure 5C,D). Tergum 10 with divided medially sensilla basiconica patch and distance between patches ca. 1.5X diameter of the patch (Figure 5C). Posterior margin of sternum 9 sclerotized, fringed with short hairs; hammer oval-shaped, slightly elevated in lateral view, surrounded by strong hairs (Figure 5E). Paraproct sclerotized heavily, with small black, hook-like tips (Figure 5F,G and Figure 6A). Aedeagus mostly membranous, basoventral area with a small patch of setae (Figure 6B,C); heart-shaped median sclerite with the surface densely covered by strong triangular spines (Figure 6E); Y-arm much longer than Y-stem, approximately 4.5× as long as Y-stem, apex of Y-arm sharp and horn-like, curved inward; lateral sclerites longer than Y-arm, approximately twice times as long as the Y-arm, gradually widen from the base to the subapical region and taper from the sub-apex to the apex (Figure 6D–F).

**Female.** Forewing length ca. 15.1 mm. Head pattern similar to male, but the spot of frons larger in male, anterior margin distinct (Figure 7A). Abdomen brown, segments 1–7 unmodified. Subgenital plate slightly produced, posterior margin with a shallow, round notch (Figure 7B).

**Distribution.** China (Liuzhou City).

**Etymology.** The specific epithet dayaoshan is derived from the Dayaoshan National Nature Reserve (Guangxi Zhuang Autonomous Region, China), the type locality where the holotype was collected. The name is used as a noun in apposition and is gender-neutral.

**Figure 8:**
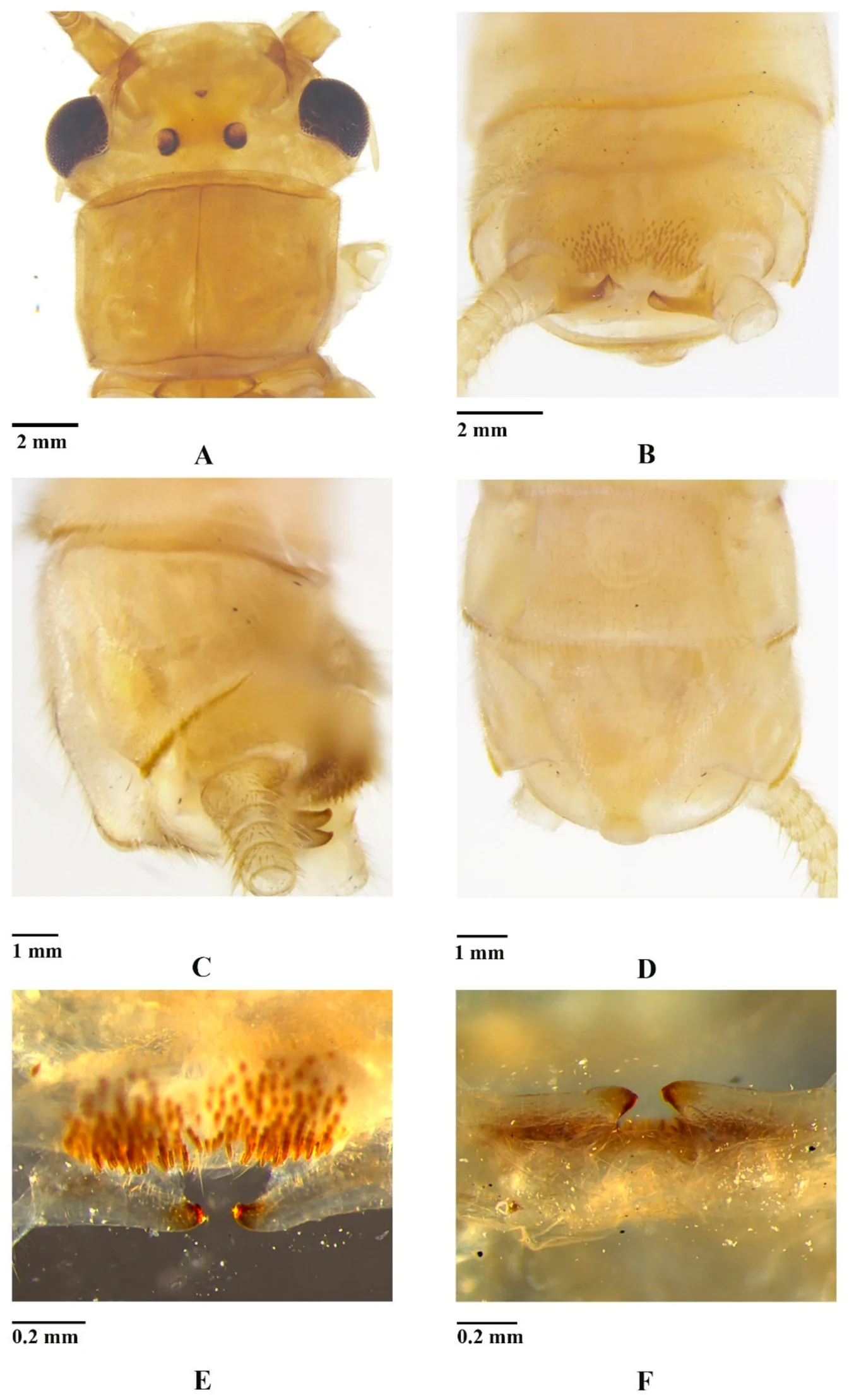
*Sinacroneuria fengyanghu* sp. nov., holotype, male, PLE038. (**A**) Head and pronotum, dorsal view; (**B**–**D**) terminalia: (**B**) dorsal view; (**C**) lateral view; (**D**) ventral view; (**E**) paraprocts, caudal view; (**F**) paraprocts, back view.

**Remarks.** The new species resembles *S. obscura* in its head pattern and terminalia. It differs from *S. obscura* in having long and slender lateral sclerites, whereas the lateral sclerites are short and stout in *S. obscura*. In addition, the median sclerite of the new species is heart-shaped, whereas it is absent in *S. obscura* and replaced by a patch of minute spines on the ventral globe (Figure 8C,E).

***Sinacroneuria fengyanghu* Y.Y. Wang, G.Q. Wang & W.H. Li sp. nov.**

**Figure 9:**
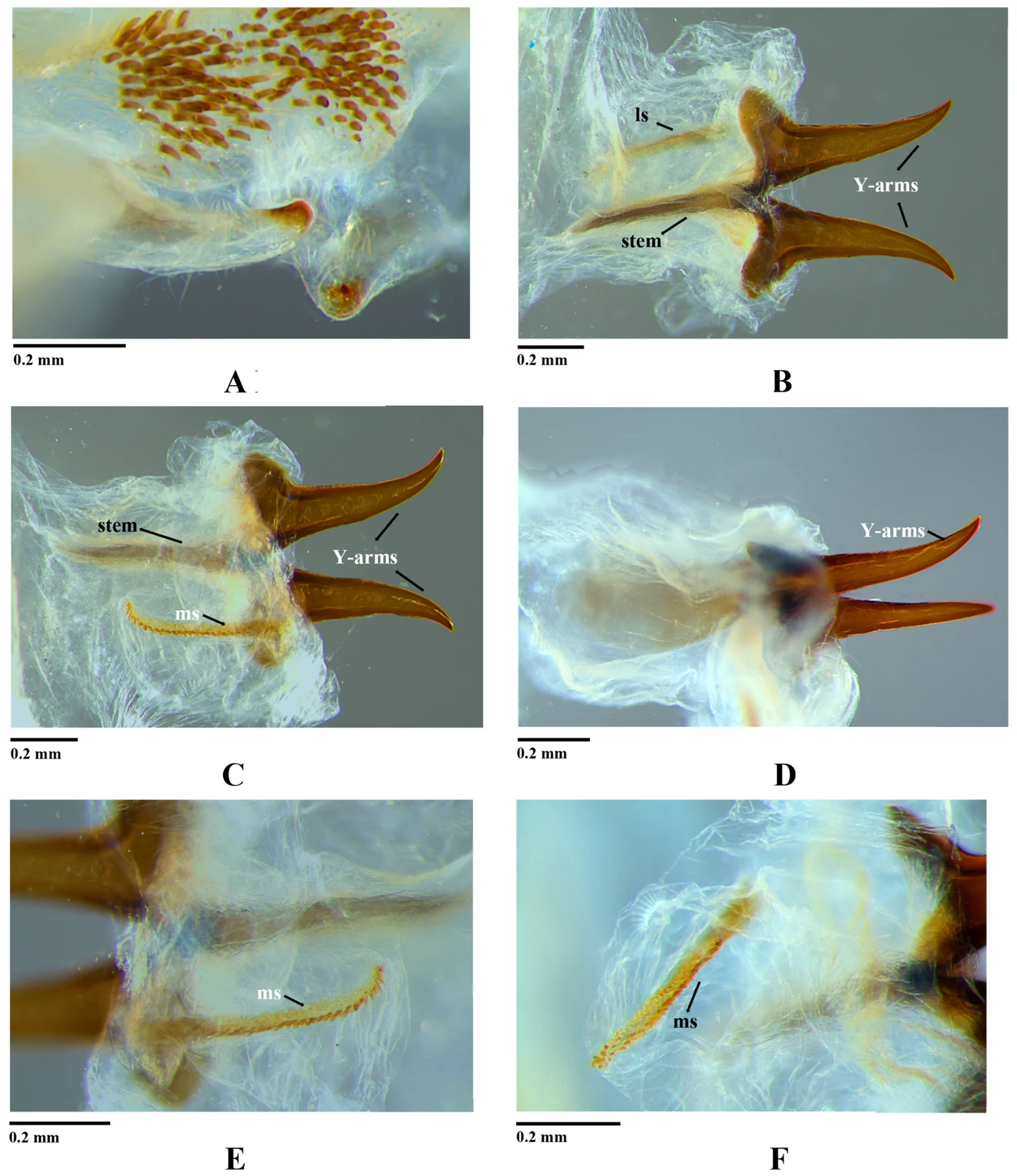
*Sinacroneuria fengyanghu* sp. nov., holotype, male, PLE038. (**A**) paraproct, dorsolateral view; (**B**–**D**) aedeagus: (**B**) dorsal view; (**C**) ventral view, (**D**). lateral view; (**E**) median sclerite, central view; (**F**) median sclerite, dorsal view.

**Figure 10:**
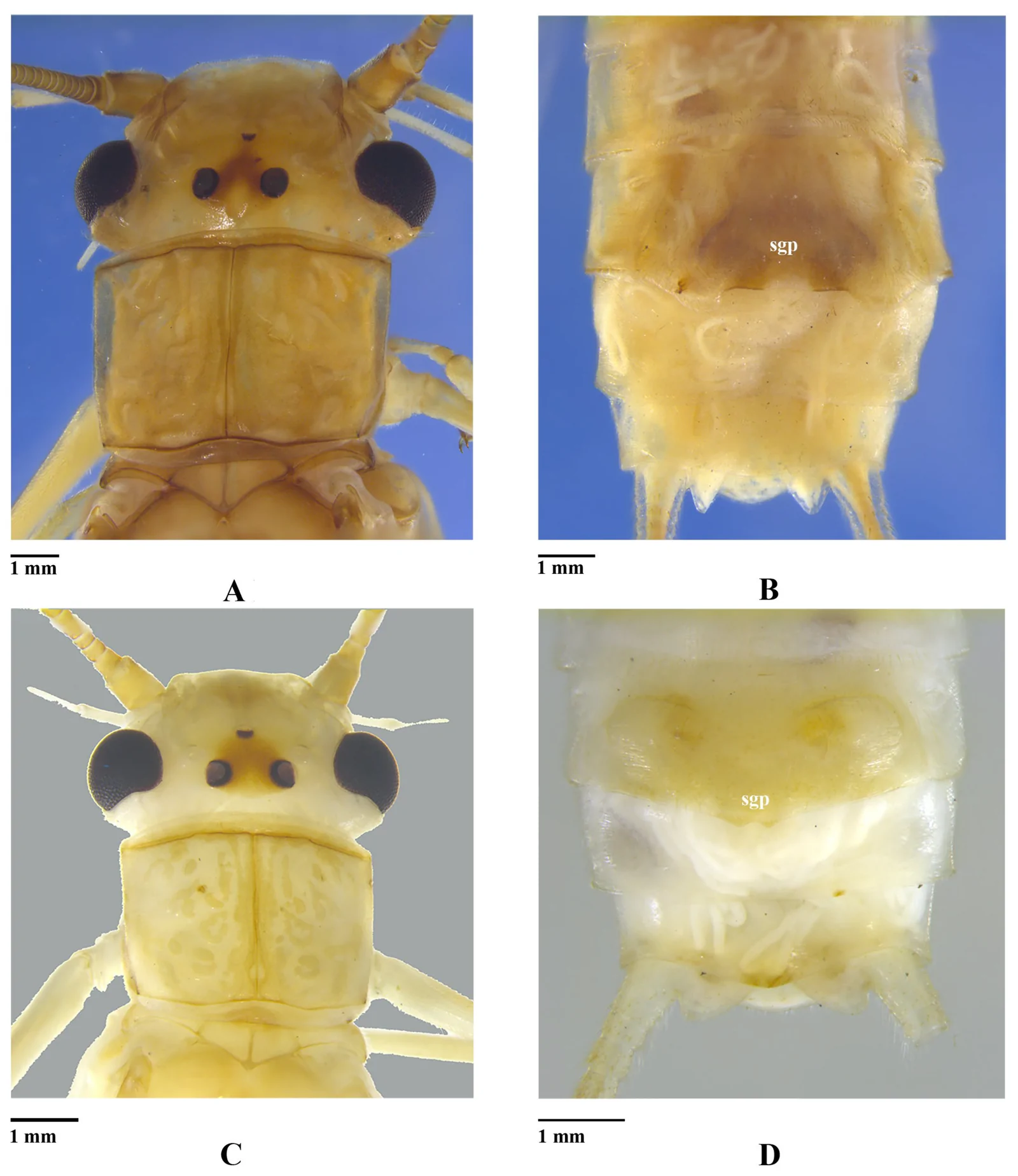
Female. (**A**,**B**) *Sinacroneuria fengyanghu* sp. nov., paratype, PLE039: (**A**) Head and pronotum, dorsal view, (**B**) terminalia, ventral view; (**C**,**D**) *Sinacroneuria longiprojecta*: (**C**) Head and pronotum, dorsal view, (**D**) terminalia, ventral view.

Chinese common name: **凤阳湖华钮𧐐**

(Figure 8, Figure 9 and Figure 10A,B)

**Specimens examined. Holotype.** ♂, CHINA: Zhejiang Province, Lishui City, Longquan City, Fengyangshan-Baishanzu National Nature Reserve, Fengyang Lake, Malaise trap, 3-VII-2007; PLE038 (HIST). **Paratypes**. 1♂, PLE039 (HIST), 1♂1♀ (NHMC), same data as holotype.

**Diagnosis.** *Sinacroneuria fengyanghu* is distinguished from congeners by the following characters: (1) triocellate, with the anterior ocellus much smaller than the lateral ocelli; (2) tergum 9 without patches of sensilla basiconica; (3) Y-arms as long as the Y-stem; (4) Y-arms with apices curved outward; (5) median sclerite long and filiform.

**Description.**

**Male.** Forewing length 13.3–13.6 mm (*n* = 3). General body color brown. Distance between ocelli much wider than the diameter of the ocellus; anterior ocellus smaller than posterior ocelli. Head with a small brown spot on ocellar triangle, anterior ocellus very small, lappets darker (Figure 8A); antennae brown. Pronotum brown with rugosities, with posterior corners rounded (Figure 8A); wing membrane clear, pale brown, with veins brown; legs mostly brown, with joint of femur and tibia and tarsi darker. Abdominal segments brown.

**Male genitalia.** Tergum 9 without sensilla patches (Figure 8B). Tergum 10 with a pair of sensilla patches, with a median notch (Figure 8B,C). Posterior margin of sternum 9 sclerotized, pale brown, fringed with short hairs; hammer oval-shaped, slightly elevated in lateral view, surrounded by short hairs (Figure 8D). Paraproct triangular, with a upcurved sharp apex, strongly sclerotized (Figure 8E,F and Figure 9A). Aedeagus mostly membranous, basoventral area with a small patch of hairs (Figure 9B,C); median sclerite long and filiform (Figure 9C,E,F); Y-arm as long as the Y-stem, apices of Y-arms curved outward, tapering to apex; lateral sclerites vague, not observed (Figure 9B,C).

**Female.** Forewing length ca. 13.1 mm. Head pattern similar to male (Figure 10A). Abdomen brown, segments 1–7 unmodified. Subgenital plate on sternum 8 slightly produced, trapezoid, posterior margin with a pair of triangular lobes with tip rounded (Figure 10B).

**Distribution.** China (Lishui City).

**Etymology.** The specific epithet fengyanghu is derived from the Fengyang Lake (Zhejiang Province, China), the type locality where the holotype was collected. The name is used as a noun in apposition and is gender-neutral.

**Remarks.** The new species is similar to *S. lii*, which is distributed in Guizhou Province and shares a similar aedeagus. It can be easily separated from *S. lii* by the small stigma on ocellar triangular which is a large spot covering ocellar triangular and a dark brown stigma on frontocylpeus area to M line in *S. lii*, the short and triangular paraproct with only tip sclerotized which is long and strongly sclerotized in *S. fengyanghu*, and the bare sensilla basiconica of tergum 9 which are with one postmedian sensilla basiconica patch, which bears a small median notch on the anterior margin.

***Sinacroneuria fujianensis* Sivec & Stark, 2020**

Chinese common name: **福建华钮𧐐**

*Sinacroneuria fujianensis* Sivec & Stark, 2020: 581, 582 [5]. HT: ♂, China, Fujian, Wuyishan City, Mt. Wuyi, Guadun; ZFMK.

Diagnosis. *Sinacroneuria fujianensis* is distinguished from congeners by the following characters: (1) triocellate; (2) Y-arm slightly shorter than sclerite Y-stem; (3) Y-arms slightly longer than Y-stem; (4) Y-arms with apices curved outward; (5) median sclerite absent.

Distribution. China: Fujian (Wuyishan City) [5].

Remarks. Specimens of *S. fujianensis* were not examined in the present study; therefore, diagnosis was based on published descriptions and type photos. Sivec and Stark [5] originally described this species from Fujian Province based on a male specimen.

***Sinacroneuria huzhengkuni* Du & Huo, 2020**

Chinese common name: **胡氏华钮𧐐**

(Figure 1D, Figure 2D and Figure 3D)

*Sinacroneuria huzhengkuni* Du & Huo, 2020: 283, 284 [11]. HT: ♂, China, Guizhou, Tongren City, Mount Fanjing.

**Specimens examined.** 1♂, China, Shaanxi Province, Hanzhong City, Foping National Nature Reserve, 33°41′24″ N, 107°54′36″ E, 12-VII-2023, 1555 m, Weihai Li, Yao Zhang, Gang Yao leg. (HIST).

**Diagnosis**. *Sinacroneuria huzhengkuni* is distinguished from congeners by the following characters: (1) triocellate, with the anterior ocellus smaller than the lateral ocelli; (2) Y-arms much longer than the Y-stem; (3) Y-arms with apices curved outward; (4) median sclerite small and triangular.

**Distribution**. China: Guizhou (Tongren City) [11], Shaanxi (Hanzhong City).

**Remarks.** Huo and Du [11] described *S. huzhengkuni* from Guizhou, China, with color images and detailed description. We document a new provincial record from Shaanxi based on a single male; the female remains unknown.

***Sinacroneuria lateralis* Sivec & Stark, 2020**

Chinese common name: **侧突华钮𧐐**

*Sinacroneuria lateralis* Sivec & Stark, 2020: 582, 583 [5]. HT: ♂, China, Fujian, Wuyishan City, Mt. Wuyi, Guadun; ZFMK.

Diagnosis. *Sinacroneuria lateralis* is distinguished from congeners by the following characters: (1) triocellate; (2) tergum 9 with a small, raised mesal lobe and a sparse patch of sensilla basiconica; (3) tergum 10 with a slightly notched anteromesal margin and a large patch of sensilla basiconica narrowly divided by a faint dark median line; (4) Y-arms about 1.3 times as long as the Y-stem, laterally compressed, with blunt apices; (5) Y-arms with apices curved outward.

Distribution. China: Fujian (Wuyishan City) [5].

Remarks. Specimens of *S. lateralis* were not examined in the present study; therefore, diagnosis was based on published descriptions and type photos. Sivec and Stark [5] originally described this species from Fujian Province based on a male specimen.

***Sinacroneuria lii* Du & Huo, 2022**

Chinese common name: **李氏华钮𧐐**

(Figure 1E, Figure 2E and Figure 3E)

*Sinacroneuria lii* Du & Huo, 2022: 284, 285 [11]. HT: ♂, China, Guizhou, Zunyi City, Suiyang County, Kuankuoshui; ICYZU.

**Specimens examined.** 1♂, China, Guizhou Province, Zunyi City, Suiyang, Kuankuoshui National Nature Reserve, Zhongxin station, 4-VI-2014, Jiancheng Lei leg. (HIST).

Diagnosis. *Sinacroneuria lii* is distinguished from congeners by the following characters: (1) triocellate; (2) Y-stem almost as long as the Y-arms; (3) Y-arms with apices curved outward; (4) long, filiform median sclerite with the dorsal surface fully covered by short spines.

Distribution. China: Guizhou (Zunyi City) [11].

Remarks. Huo and Du [11] described *S. huzhengkuni* from Guizhou Province. The male examined in the present study was collected at the type locality and agrees fully with the diagnostic morphology of the holotype

***Sinacroneuria longiprojecta* Du & Huo, 2022**

Chinese common name: **长突华钮𧐐**

(Figure 1F, Figure 2F, Figure 3F and Figure 10C,D)

*Sinacroneuria longiprojecta* Du & Huo, 2022: 490, 491 [10]. HT: ♂, China, Hangzhou City, Tianmu Mountain; ICYZU.

**Specimens examined.** 1♂2♀, China, Zhejiang Province, Hangzhou City, Lin’an District, Western Tianmushan, Tianmu Mountain National Nature Reserve Administration Bureau, 300 m, 28-VI-2013, Xiao Zhang leg. (CAUC); 1♂, China: Zhejiang Province, Tianmu Mountain, Chanyuan Temple, Fasheng Li leg. (CAUC).

Diagnosis. *Sinacroneuria longiprojecta* is distinguished from congeners by the following characters: (1) triocellate, with the anterior ocellus smaller than the lateral ocelli; (2) Y-stem about one-third the length of the Y-arms; (3) Y-arms with apices curved outward; (4) lateral sclerites sclerotized basally, about twice as wide as the Y-arms, with pointed apices; (5) median lobe absent.

Female (Figure 10C,D). Forewing length ca. 17.2 mm. Sternum 8 slightly sclerotized, with a pair of large, reniform sclerites; subgenital plate emarginate, with lobes projecting posteriorly, reaching half the length of sternum 9.

Distribution. China: Zhejiang (Hangzhou City) [10].

Remarks. Xiang et al. described *S. longiprojecta* from Zhejiang Province, China, with color photographs and a detailed description; the female, however, has not previously been documented [10]. This study documents the female specimens of this species for the first time, revealing several distinctive morphological characteristics in females.

***Sinacroneuria longwangshana* (Yang & Yang, 1998)**

Chinese common name: **龙王山华钮𧐐**

(Figure 1G, Figure 2G and Figure 3G)

*Acroneuria longwangshana* Yang & Yang, 1998: 40 [4]. HT: ♂, China, Zhejiang, Huzhou City, Anji County, Longwang Mount; CAUC.

*Sinacroneuria longwangshana*: Du et al., 2001: 74 [12]; Li et al., 2014: 288 [6].

Diagnosis. *Sinacroneuria longwangshana* is distinguished from congeners by the following characters: (1) triocellate, with the anterior ocellus smaller than the lateral ocelli; (2) Y-stem slightly shorter than the Y-arms; (3) Y-arms with apices curved outward; (4) median sclerite shorter than the Y-arms.

Distribution. China: Zhejiang (Huzhou City) [4].

Remarks. This species was originally described in 1998 based on two male specimens from Zhejiang, China, with illustrations and a detailed description.

***Sinacroneuria magnimaculata* Y.Y. Wang, G.Q. Wang & W.H. Li sp. nov.**

**Figure 11:**
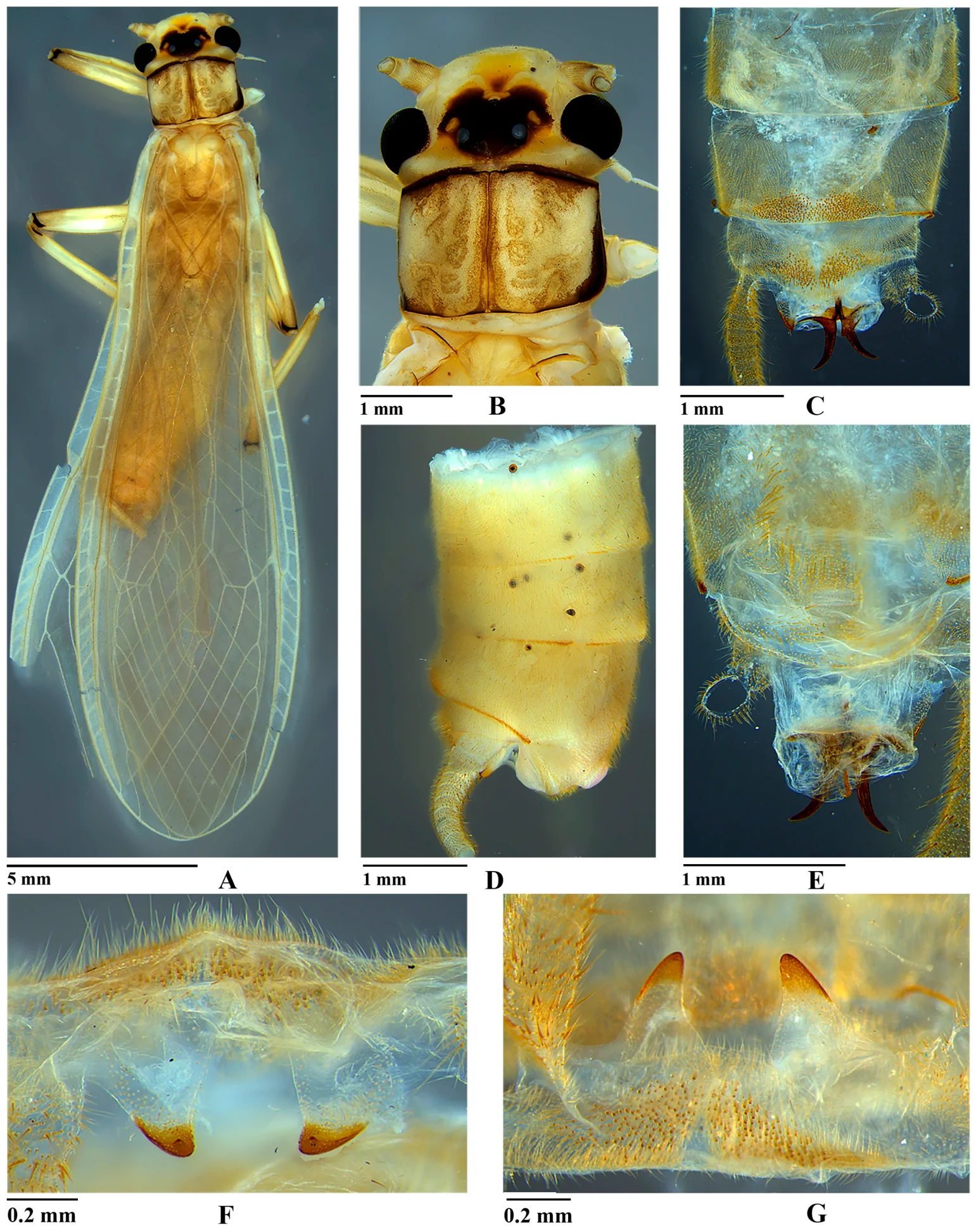
*Sinacroneuria magnimaculata* sp. nov., holotype, male, PLE040. (**A**) male habitus, dorsal view; (**B**) Head and pronotum, dorsal view; (**C**–**E**) terminalia: (**C**) dorsal view; (**D**) lateral view; (**E**) ventral view; (**F**) paraprocts, caudal view; (**G**) paraprocts, back view.

**Figure 12:**
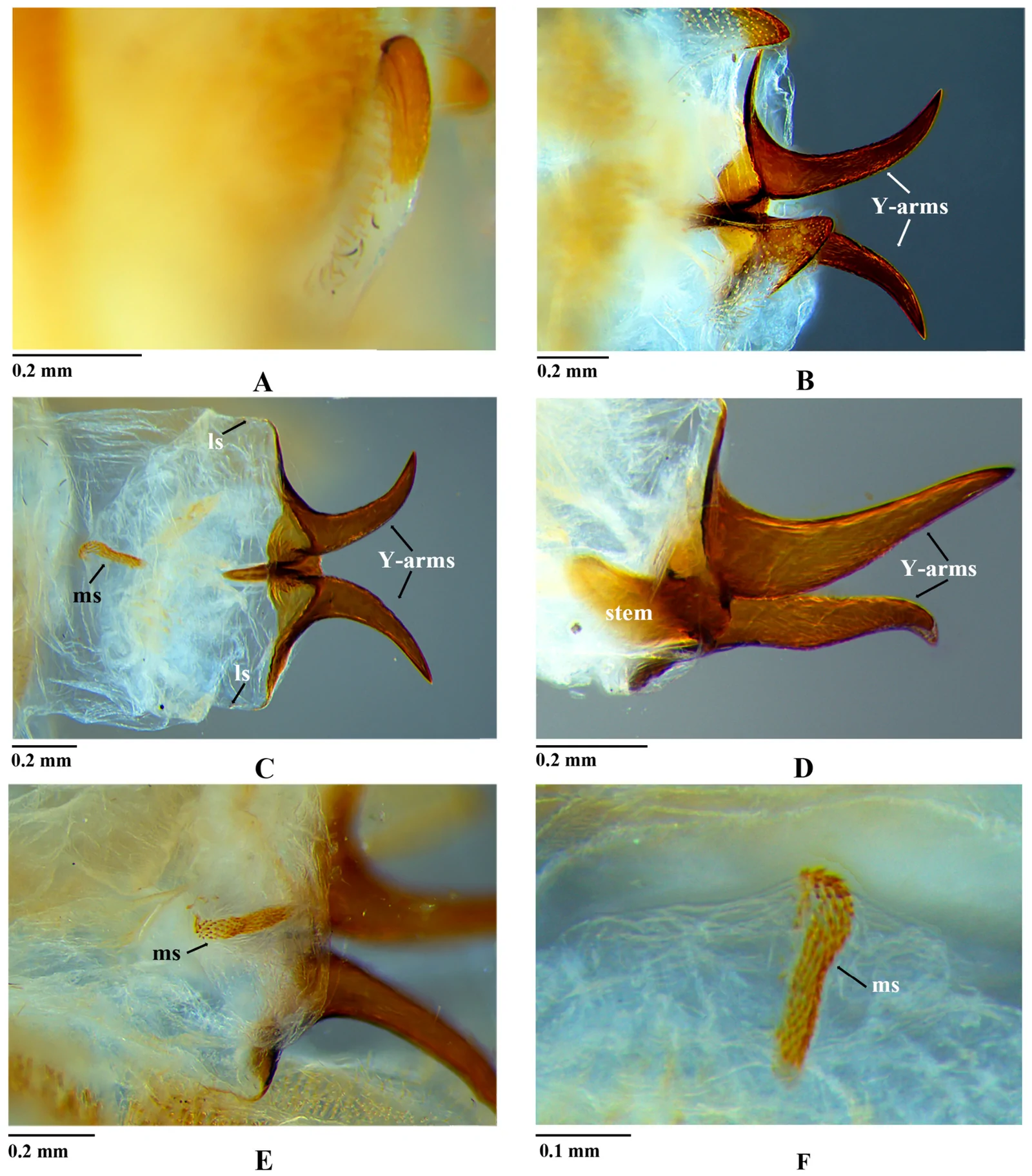
*Sinacroneuria magnimaculata* sp. nov., holotype, male, PLE040. (**A**) paraproct, dorsolateral view; (**B**–**D**) aedeagus: (**B**) dorsal view; (**C**) ventral view, (**D**). lateral view; (**E**) median sclerite, ventral view; (**F**) median sclerite, ventral view.

Chinese common name: **大斑华钮𧐐**

(Figure 11 and Figure 12)

**Specimens examined. Holotype.** 1♂, China, Anhui Province, Huangshan City, Tangkou Town, Qingxijingyuan, Light, 541 m, 30°4′34″ N, 118°9′20″ E, 28-VII- 2023, Yizhen Han leg.; PLE040 (HIST).

**Diagnosis.** *Sinacroneuria magnimaculata* is distinguished from congeners by the following characters: (1) triocellate; (2) Y-arms about 2 times as long as the Y-stem; (3) Y-arms with apices curved outward; (4) median sclerite longer than the lateral sclerites, with the dorsal surface covered by short spines.

**Description.**

**Male**. Forewing length ca. 19.8 mm. General body color light brown. Distance between posterior ocelli much wider than diameter of the one posterior ocellus. Head light brown, with a small brown spot on the anterior frons and a large dark brown spot covering the ocelli and tentorial callosities; lappets pale; M-line pale (Figure 11A,B); Pronotum brown, with lighter rugosities, margins dark (Figure 11A,B). Wing membrane translucent, with veins pale brown; legs mostly light brown, with femorotibial joint dark brown. Abdominal segments pale brown.

**Male genitalia.** Tergum 9 with a wide postmedian sensilla basiconica patch; a small median notch occurs on the anterior margin; tergum 10 patch narrowly but completely divided (Figure 11C,D). Hammer pale pink, oval and slightly plump, set near posterior margin of sternum 9, with fine rugosities on surface (Figure 11E). Paraproct triangular and pale, broad basally, apex tapering and tip obtuse in dorsal view, only tips heavily sclerotized and brown, with a tiny dorsoapical spine (Figure 11F,G and Figure 12A).

Aedeagus. Apical lobe with a bristle ring (Figure 12B,C). Apex of Y-arm sharp and horn-like, curved outward and upward, Y-arm about 2 times as long as the Y-stem (Figure 12B–D); lateral sclerites short, slender and slightly sclerotized, truncated terminally (Figure 12C,D); the median sclerite longer than the lateral sclerites and filiform, dorsal surface covered by short spines (Figure 12E,F).

**Female**. Unknown.

**Distribution**. China (Anhui Province).

**Etymology.** The specific epithet magnimaculata is derived from Latin magnus (large) and maculatus (spotted), referring to the large dark marking on the head.

**Remarks.** The new species is most similar to *Sinacroneuria obscura* Li and Murányi, 2017 from Guangxi, China, in sharing a similar patch of sensilla basiconica and similar aedeagal sclerites. It can be easily separated by the presence of a subtriangular ventral sclerite connected to the Y-shaped aedeagal structure, which is absent in *S. obscura*. In addition, the paraproct of the new species is broadly triangular basally, with only the apical portion heavily sclerotized and brown, bearing a tiny dorsoapical spine, whereas that of *S. obscura* is slender, entirely sclerotized, and claw-like (Figure 2G, Figure 3G and Figure 12).

**Sinacroneuria obscura Li & Murányi, 2017**

Chinese common name: 弱眼华钮𧐐

(Figure 1H, Figure 2H and Figure 3H)

*Sinacroneuria obscura* Li & Murányi, 2017: 96–102 [7]. HT: ♂, China, Guangxi Zhuang Autonomous Region, Fangchenggang City, Shiwandashan; HIST.

**Specimens examined.** Holotype. ♂, China: Guangxi Zhuang Autonomous Region, Fangchenggang City, Shangsi County, Shiwandashan National Forest Park, Pearl River above tourist route bridge, 375 m, 21°59′24″ N, 107°54′0″ E, Jenő Kontschan, Junyi Li, Shan Li, Weihai Li, Dávid Murányi, Guoquan Wang leg, (HIST); 1♂, China, Guangxi Zhuang Autonomous Region, Fangchenggang City, Shiwandashan National Forest Park, 17-V-2023, Xingyue Liu leg. (CAUC).

Diagnosis. *Sinacroneuria obscura* is distinguished from congeners by the following characters: (1) biocellate, with the vestigial anterior ocellus represented by a small dark spot; (2) Y-arms about 3 times as long as the Y-stem, with the basal portions nearly parallel-sided and apices pointed; (3) Y-arms with apices curved outward; (4) median sclerite absent.

Distribution. China: Guangxi (Fangchenggang City) [7].

Remarks. This species was originally described in 2017 based on male and female specimens from Guangxi Zhuang Autonomous Region, China, with color image and detailed description.

***Sinacroneuria orientalis* Yang & Yang, 1995**

Chinese common name: 东方华钮𧐐

(Figure 1I, Figure 2I and Figure 3I)

*Sinacroneuria orientalis* Yang & Yang, 1995: 64 [3]. HT: ♂, China, Anhui, Huangshan; CAUC.

*Kamimuria flavata* Navás, 1933: 82 [13]; Wu 1938: 54 [13]; Sivec & Stark, 2020: 581 [5].

*Sinacroneuria flavata*: Du et al., 1999: 64 [14] (synonymized by Sivec and Stark [5]).

*Sinacroneuria orientalis*: Liu et al., 2025: 81 [2].

**Specimens examined.** Holotype. ♂, China, Anhui Province, Huangshan City, Huangshan, 17/23-VII-1997, Jikun Yang leg. (CAUC).

**Diagnosis**. *Sinacroneuria orientalis* is distinguished from congeners by the following characters: (1) triocellate; (2) Y-arms almost as long as the Y-stem; (3) Y-arms with apices curved outward; (4) median lobe absent.

**Distribution**. China: Anhui (Huangshan City) [3], Zhejiang (Hangzhou City) [5].

**Remarks**. Navás described *Perla (Kamimuria) flavata* from Japan, and later Navás described *Kamimuria flavata* from China [13,15]. The latter name is an invalid secondary homonym of *Kamimuria flavata* Navás, 1924 and is unavailable under the ICZN. Based on similarities in the female subgenital plate and head pattern, *Kamimuria flavata* Navás, 1933 is considered conspecific with *Sinacroneuria orientalis* Yang and Yang, 1995. However, because the former name cannot be used after transfer to *Sinacroneuria*, *S. orientalis* remains the valid name.

***Sinacroneuria parallela* Xiang & Du, 2022**

Chinese common name: **平行华钮𧐐**

(Figure 1J, Figure 2J and Figure 3J)

*Sinacroneuria parallela* Xiang & Du, 2022: 488 [10]. HT: ♂, China, Zhejiang, Fengyang Mount; ICYZU.

**Specimen examined.** 1♂, China, Fujian Province, Nanping City, Xingcun Town, Mt. Wuyi, Guadun, 17-VI-2026, Raorao Mo, Jianjun Huang leg. (HIST).

**Diagnosis**. *Sinacroneuria parallela* is distinguished from congeners by the following characters: (1) triocellate, with the anterior ocellus smaller than the lateral ocelli; (2) Y-arms about 2 times as long as the Y-stem; (3) Y-arms nearly straight and parallel to each other; (4) Y-arms with apices curved inward; (5) median sclerite nearly as long as the Y-arms.

**Distribution**. China: Zhejiang (Nanping City) [10].

**Remarks**. This species was originally described in 2022 based on a single male from Zhejiang, China [11].

***Sinacroneuria quadriplagiata* (Wu, 1938)**

Chinese common name: **四斑华钮𧐐**

(Figure 1K, Figure 2K and Figure 3K)

*Acroneuria quadriplagiata* Wu 1938*:* 134 [16]. HT: ♂, China, Zhejiang Province, Tianmu Mount; Lost; Illies, 1966: 311;

*Sincroneuria quadriplagiata*: Du et al., 2001: 74 [12]; Li et al., 2014: 288, 289 [6].

**Specimens examined.** 1♂, China, Zhejiang Province, Lishui City, Fengyang Mountain, Huangmaojian, 30-VII-2007, Jingxian Liu leg. (CAUC). 

**Diagnosis**. *Sinacroneuria quadriplagiata* is distinguished from congeners by the following characters: (1) triocellate, with the anterior ocellus smaller than the lateral ocelli; (2) Y-stem shorter than the Y-arms; (3) Y-arms with apices curved outward; (4) median sclerite with several apical spines. 

**Distribution**. China: Zhejiang (Hangzhou City [5,16], Lishui City).

**Remarks**. Although the holotype of *S. quadriplagiata* has been lost and only the original description by Wu [13] is available, the examined specimens from Lishui, Zhejiang Province, approximately 276 km from the type locality (Tianmushan), are provisionally assigned to this species based on the available diagnostic characters. The examined specimens generally agree with the original description and subsequent taxonomic treatments, particularly in the pronotal markings, the paired groups of sensilla basiconica on male terga 9 and 10, and the dark, bluntly pointed, upcurved paraprocts. Although topotypic material is unavailable, we found no morphological evidence that clearly contradicts their assignment to *S. quadriplagiata*. We therefore retain this identification pending the examination of additional material, especially topotypic specimens.

***Sinacroneuria similiwui* Y.Y. Wang, G.Q. Wang & W.H. Li sp. nov.**

**Figure 13:**
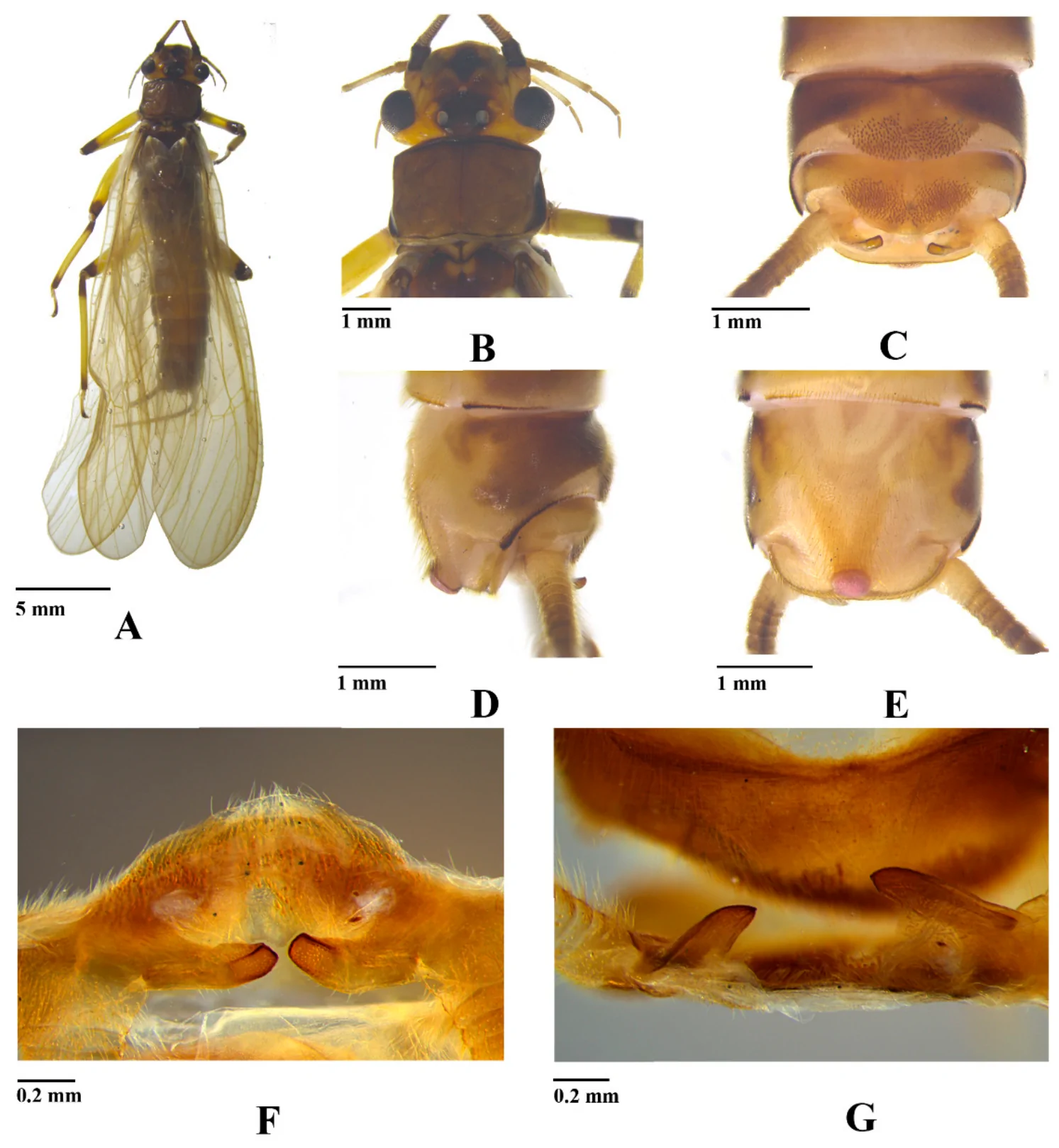
*Sinacroneuria similiwui* sp. nov., holotype, male, PLE041. (**A**) male habitus, dorsal view; (**B**) Head and pronotum, dorsal view (**C**–**E**) terminalia: (**C**) dorsal view; (**D**) lateral view; (**E**) ventral view; (**F**) paraprocts, caudal view; (**G**) paraprocts, back view.

**Figure 14:**
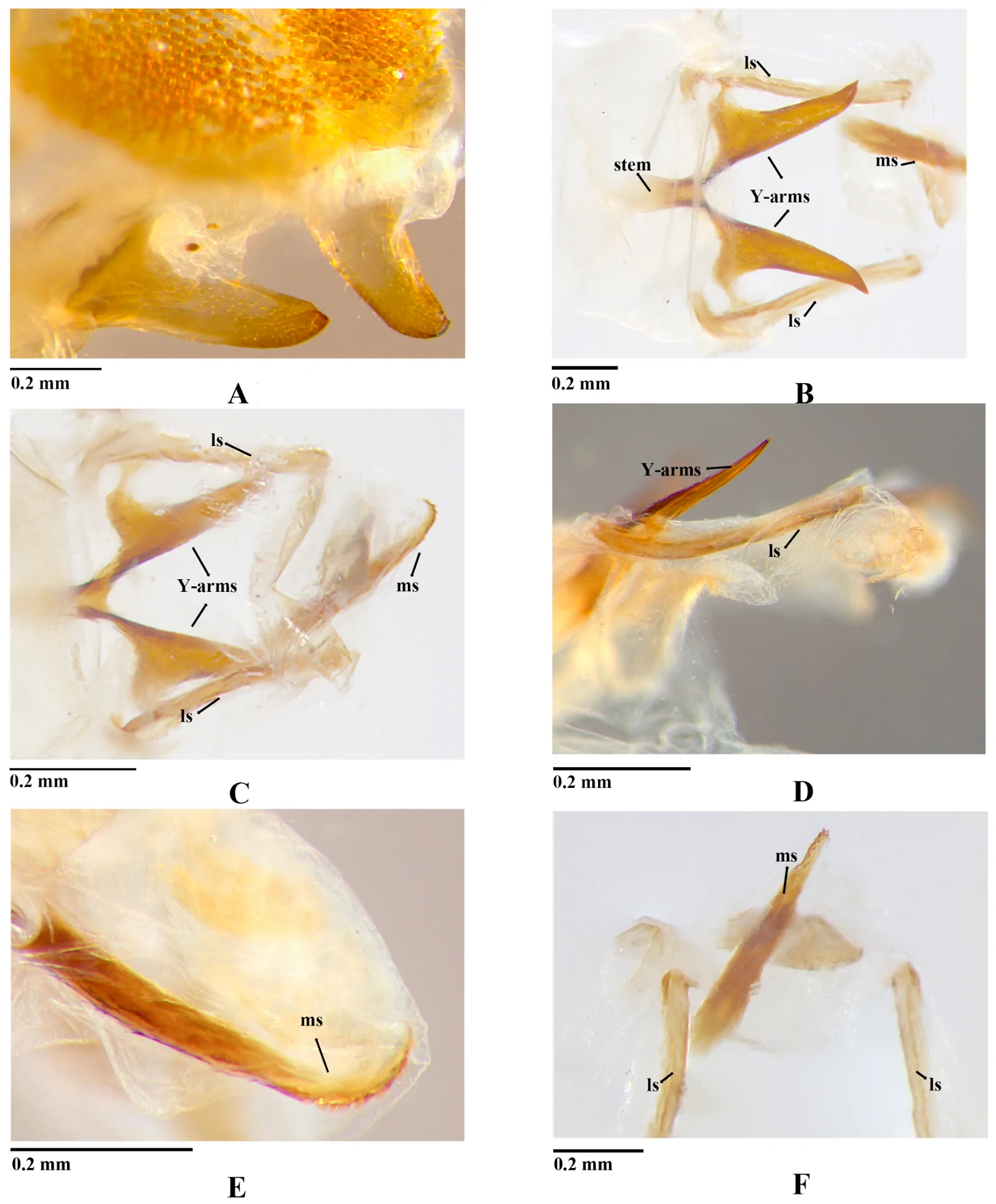
*Sinacroneuria similiwui* sp. nov., holotype, male, PLE041. (**A**) paraproct, dorsolateral view; (**B**–**D**) aedeagus: (**B**) dorsal view; (**C**) ventral view, (**D**) lateral view; (**E**) median sclerite, lateral view; (**F**) median sclerite, dorsal view.

Chinese common name: 拟吴华钮𧐐

(Figure 13 and Figure 14)

**Specimens examined. Holotype.** ♂, China, Zhejiang Province, Lishui City, Jingning County, Shangbiao Village, 1011 m, 27°42′42″ N, 119°36′35″ E, 8-V-2021, Chaoyang Kong leg.; PLE041 (HIST).

**Diagnosis.** *Sinacroneuria similiwui* is distinguished from congeners by the following characters: (1) triocellate; (2) Y-arms about 2 times as long as the Y-stem; (3) Y-arms with apices curved outward; (4) median sclerite broad basally, with an acute apex

**Description.** Male. Forewing length 22.1 mm. General body color brown. Distance between ocelli much wider than the diameter of the ocellus. Head with a small brown spot on the anterior frons and a dark brown spot covering ocellar triangle; lappets pale brown (Figure 13A); scape and pedicel dark brown, flagellum lighter. Pronotum brown with rugosities, lateral margins dark (Figure 13A); wing membrane clear, light brown, with veins brown; legs mostly brown, with joint of femur and tibia, distal tibia and tarsi dark brown. Abdominal segments dark brown.

Basal half of tergum 9 with a pair of triangular sclerites, with one elliptic sensilla patches posteriorly, posterior margins black (Figure 13B).

**Male genitalia.** Tergum 10 with a pair of sensilla basiconica patches, but the median still in contact (Figure 13B,C). Posterior margin of sternum 9 sclerotized, fringed with short hairs; hammer oval-shaped and anterior with a V-shaped sclerite, slightly elevated in lateral view, surrounded by strong hairs (Figure 13D). Paraproct triangular, sclerotized strongly, with small black, hook-like tips (Figure 13E,F and Figure 14A). Aedeagus mostly membranous, basoventral area with a small patch of hairs; median sclerite with broad base and acute tip, apex covered by strong triangular spines (Figure 14B,C); Y-arm about 2× longer than Y-stem, apices of Y-arms curved outward; lateral sclerites longer than Y-arm, ca. 1.5× length of the Y-arm, basal curved inward and distal slender (Figure 14B–D).

**Female.** Unknown.

**Distribution.** China (Lishui City).

**Etymology.** The specific epithet similiwui is derived from the Latin prefix simili- (similar) and the specific name of the closely related species *S. wui*. The name is used as a noun in apposition and is gender-neutral.

**Remarks.** The new species is similar to *Sinacroneuria wui*, which is distributed in Zhejiang Province and the Guangxi Zhuang Autonomous Region, by sharing similar genitalia (Figure 2N). It can be easily separated from *S. wui* by the stout and short Y-stem of the aedeagus, which is long and slender in the latter; the wide Y-arm base, which is narrow in *S. wui*; and the markedly short connection between the lateral sclerites and the Y-arm, which extends well beyond half the length of the Y-arm in the latter (Figure 3N and Figure 14B). The Y-arms are approximately twice as long as the Y-stem in the new species compared with approximately 1.5 times as long in *S. wui* (Figure 3N and Figure 14B). The patches of sensilla basiconica on terga 9–10 lack a pale median spot which is present in *S. wui*, and the posterior margin of tergum 10 is smoothly rounded, not bilobed with two distinct corners as in the latter (Figure 2N and Figure 14B).

***Sinacroneuria sinica* (Yang & Yang, 1998)**

**Figure 15:**
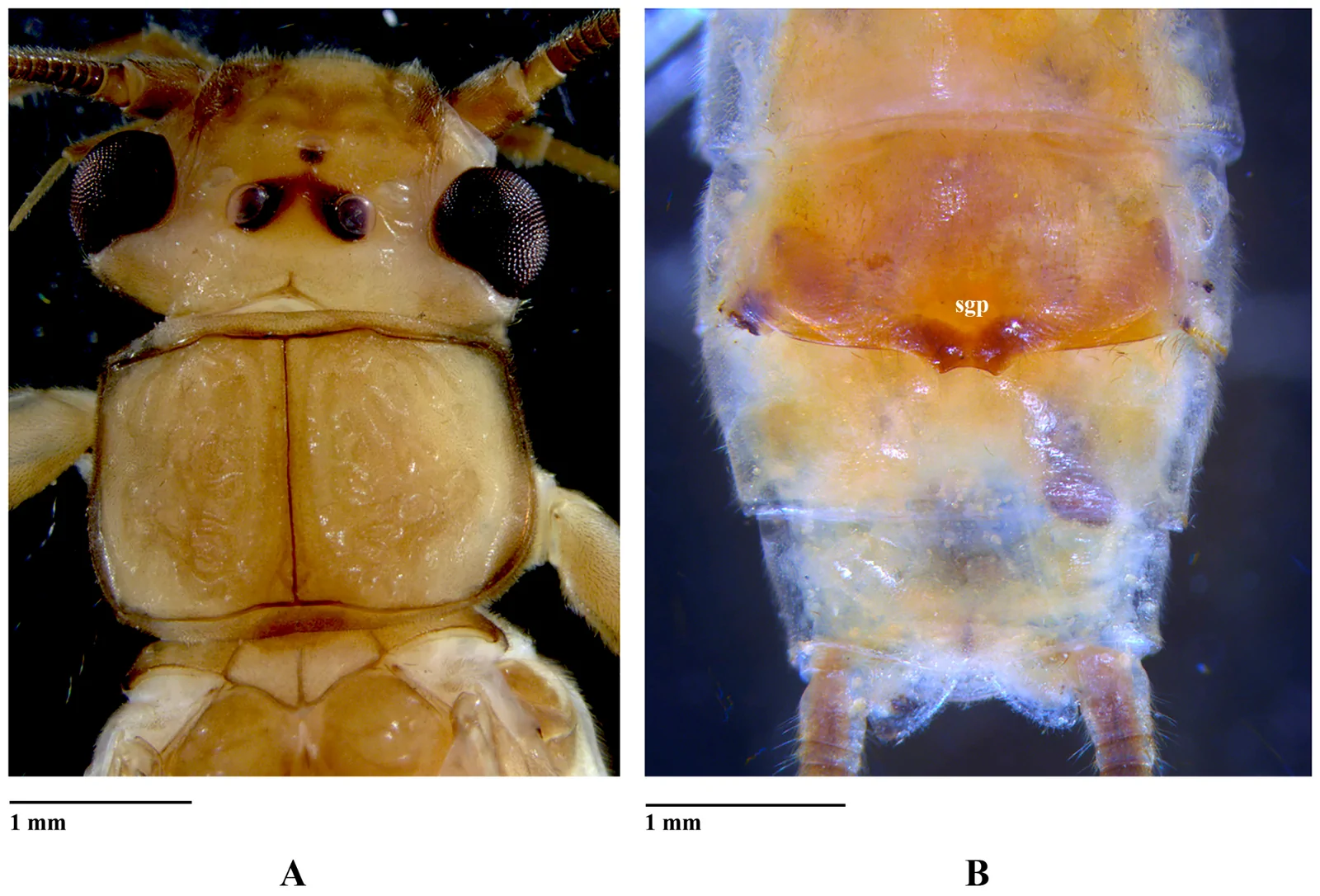
*Sinacroneuria sinica*, female. (**A**) Head and pronotum, dorsal view; (**B**) terminalia, ventral view.

Chinese common name: 中华华钮𧐐

(Figure 1L, Figure 2L, Figure 3L and Figure 15)

*Acroneuria sinica* Yang & Yang, 1998: 41. 2 [4]. HT: ♂, China, Zhejiang, Huzhou City, Anji County, Longwangshan; CAUC.

*Sinacroneuria sinica* (Yang & Yang, 1998): Murányi & Li, 2016: 180 [9]; Liu, Mo & Li, 2019: 371 [17].

**Specimens examined.** Holotype. ♂, China, Zhejiang Province, Huzhou City, Anji County, Longwangshan, 10-VIII-1995, Hong Wu leg. (CAUC). Paratype: 1 ♂, same locality as holotype, 12-VI-1996 (CAUC); Additional material examined. 2♂2♀, China, Fujian Province, Wuyishan City, Mt. Wuyi, 27°45′0″ N, 117°40′48″ E, 735 m, 12-VII-2009, Shi Li, Xiaoyan Liu leg. (CAUC).

Diagnosis. *Sinacroneuria sinica* is distinguished from congeners by the following characters: (1) triocellate, with the anterior ocellus smaller than the lateral ocelli; (2) Y-arms about 2 times as long as the Y-stem; (3) Y-arms with apices curved outward; and (4) median sclerite shorter than the Y-arms, slender with a globular base, and apically covered with spinules.

Female (Figure 15). Forewing length 19.2–20.1 mm (*n* = 2). Sternum 8 broadly trapezoidal, sclerotized, with posterior area darker; the subgenital plate is medially emarginate and extends posteriorly to the anterior margin of sternum 9.

Distribution. China: Zhejiang (Huzhou City) [4], Fujian (Wuyishan City).

Remarks. This species was originally described in 1998 based on male specimens from Zhejiang Province, China [4]. We examined male and female specimens from Fujian Province, which represent the new provincial record for this species.

***Sinacroneuria transversa*** **Liu, Cao & Li, 2025**

Chinese common name: 横斑华钮𧐐

(Figure 1M, Figure 2M and Figure 3M)

*Sinacroneuria transversa* Liu, Cao & Li, 2025: 86 [2]. HT: ♂, China, Fujian, Wuyishan City, Mt. Wuyi, Kekao station; CAUC.

**Specimens examined.** Holotype. ♂, China, Fujian Province, Wuyishan City, Mt. Wuyi, Kekao station, 27°44′34″ N, 117°40′27″ E, 12-VII-2009, 735 m, Light, Shi Li, Xiaoyan Liu leg. (CAUC). Paratype: 1 ♂; Zhejiang Province, Lishui city, Fengyangshan; 27°55′ N, 119°13′ E; 1178 m; 30-VII-2007; Jingxian Liu leg. (HIST).

Diagnosis. *Sinacroneuria transversa* is distinguished from congeners by the following characters: (1) triocellate, with the anterior ocellus smaller than the lateral ocelli; (2) Y-arms about 3 times as long as the Y-stem; (3) Y-arms with apices curved outward; (4) crescent-shaped median sclerite.

Distribution. China: Fujian (Wuyishan City), Zhejiang (Lishui City) [2].

Remarks. This species was originally described in 2025 based on male specimens from Fujian Province, China.

***Sinacroneuria wui* (Yang & Yang, 1998)**

Chinese common name: 吴氏华钮𧐐

(Figure 1N, Figure 2N and Figure 3N)

*Sinacroneuria wui* (Yang & Yang, 1998): 42 [4]. HT: ♂, China, Zhejiang Province, Anji City, Longwangshan; CAUC.

*Acroneuria wui* Yang & Yang, 1998: 42 [4].

*Sinacroneuria wui*: Li, Murányi & Wang, 2014: 290 [6]; Murányi & Li, 2016: 185 [9]; Mo, Wang, Yang, Li & Murányi, 2022: 19 [18].

**Specimens examined.** Holotype. ♂, China, Zhejiang Province, Anji City, Longwangshan, 12-VI-1996, Hong Wu leg. (CAUC).

**Distribution**. China: Guangdong (Shaoguan) [16], Guangxi (Guilin) [16], Zhejiang (Anji) [4].

**Diagnosis**. *Sinacroneuria wui* is distinguished from congeners by the following characters: (1) triocellate; (2) Y-arms almost as long as the Y-stem; (3) Y-arms with apices curved outward; (4) median sclerite longer than the Y-arms, broad basally and tapering toward the apex.

**Remarks**. This species was originally described in 1998 based on a single male specimen from Zhejiang Province, China. Mo et al. [16] provided new provincial records based on specimens from Guangxi and Guangdong Province. In this study, we examined those specimens and another male specimen from Lishui City in Zhejiang Province.

***Sinacroneuria yiui* (Wu, 1935)**

Chinese common name: 四川华钮𧐐

*Mesoperla yiui* Wu, 1935: 230 [19]. HT: ♂, China, Jiangxi Province, Jiujiang City, Lushan; Lost.

*Acroneuria yiui*: Wu, 1938: 135 [16]; Yang & Yang, 1998: 42 [4].

*Sinacroneuria yiui*: Du et al., 2001: 75 [12]; Li, Murányi & Wang, 2014: 290 [6]; Muranyi & Li, 2016: 185 [9].

Diagnosis. *Sinacroneuria yiui* is distinguished from congeners by the following characters: (1) triocellate; (2) Y-arms about 2 times as long as the Y-stem; (3) Y-arms with apices curved inward; (4) short, claviform median sclerite.

Distribution. China: Jiangxi (Jiujiang City) [19], Zhejiang [16,19].

Remarks. Wu [15] described this species as *Mesoperla yiui* based on a Jiangxi male holotype, but the type specimen was lost during World War II; Wu [18] supplied a female description and transferred this species into *Acroneuria*, with a new record in Zhejiang Province. The species was subsequently transferred to *Sinacroneuria* by Du et al. [19].

***Sinacroneuria yunjishan* Y.Y. Wang, G.Q. Wang & W.H. Li sp. nov.**

**Figure 16:**
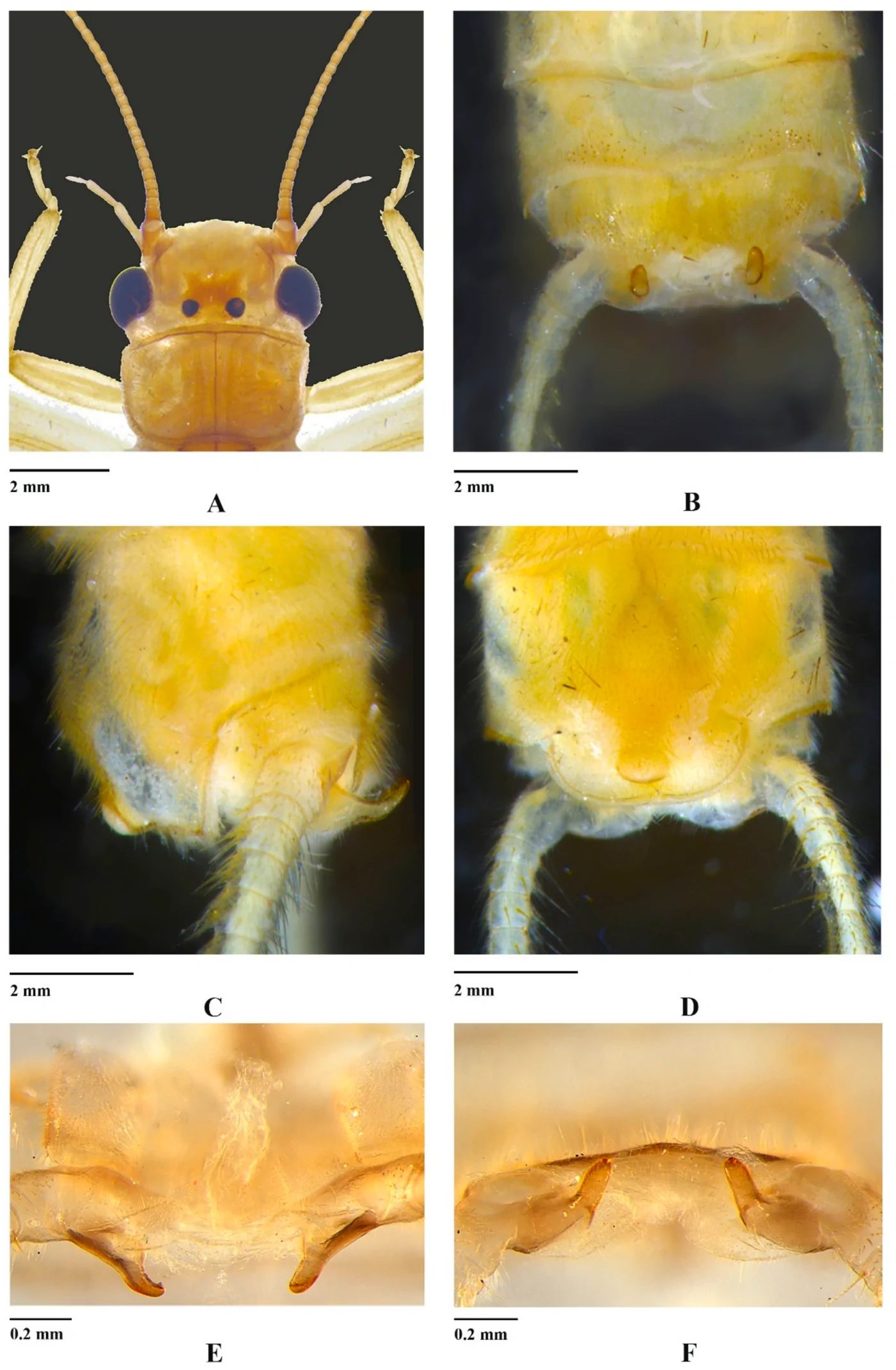
*Sinacroneuria yunjishan* sp. nov., holotype, male, PLE042. (**A**) Head and pronotum, dorsal view; (**B**–**D**): terminalia (**B**) dorsal view; (**C**) dorsal view; (**D**) ventral view; (**E**) paraprocts, caudal view; (**F**) paraprocts, back view.

**Figure 17:**
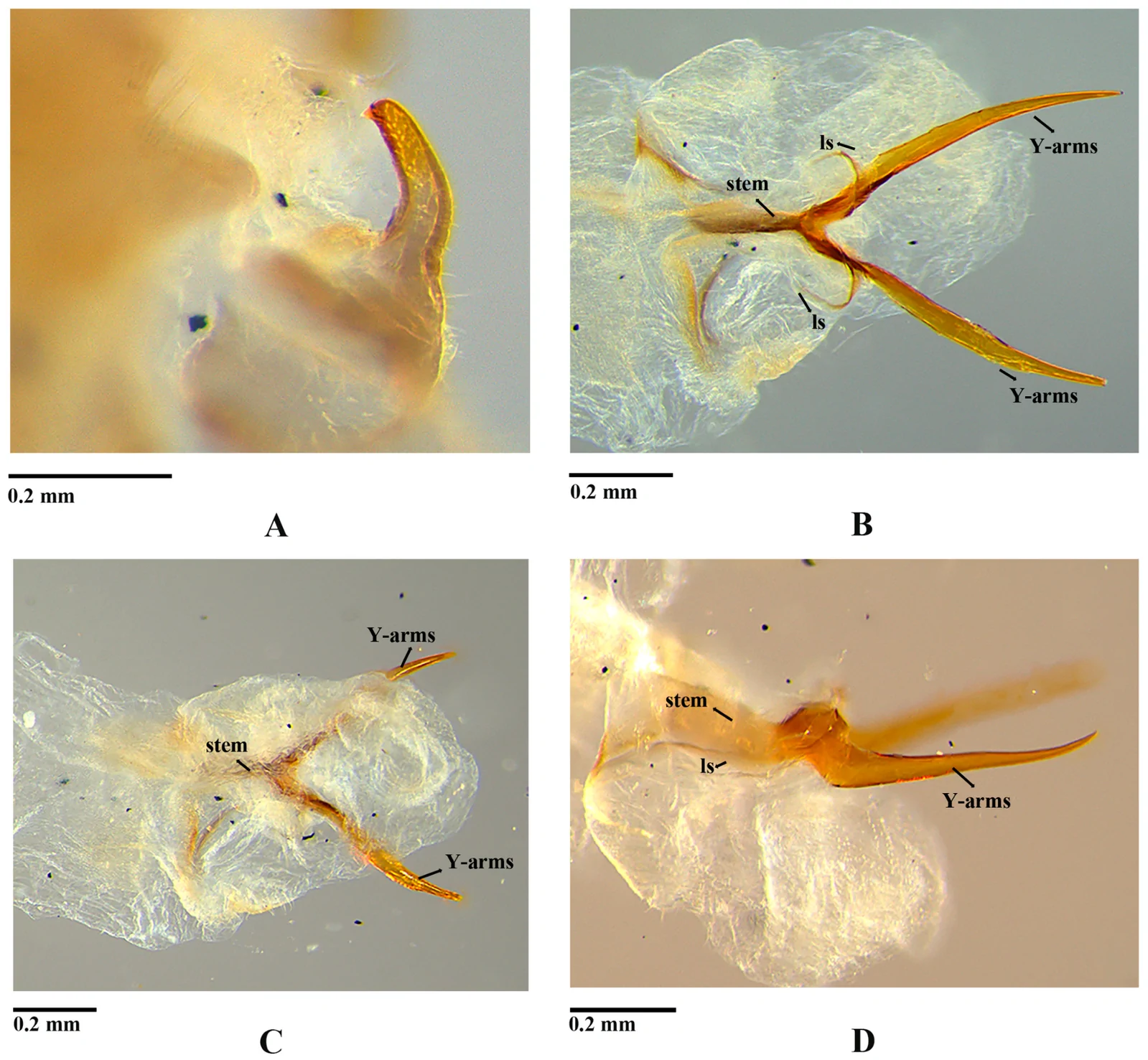
*Sinacroneuria yunjishan* sp. nov., holotype, male, PLE042. (**A**) paraproct, dorsolateral view; (**B**–**D**) aedeagus: (**B**) dorsal view; (**C**) ventral view; (**D**). lateral view.

Chinese common name: **云髻山华钮𧐐**

(Figure 16 and Figure 17)

**Specimens examined. Holotype.** ♂, China, Guangdong Province, Shaoguan City, Xinfeng County, Yunjishan, 19-VII-2003, Shuwen An. leg.; PLE042 (HIST).

**Diagnosis.** *Sinacroneuria yunjishan* is distinguished from congeners by the following characters: (1) biocellate; (2) tergum 10 with few sensilla basiconica posteriorly; (3) Y-arms about 3 times as long as the Y-stem; lateral sclerites long and slender; (4) Y-arms with apices curved inward; (5) median sclerite absent.

**Description.**

**Male.** General body color brown. Forewing length ca. 25.8 mm. Distance between ocelli much wider than the diameter of the ocellus. Head with a small brown spot on the anterior frons and a dark brown spot covering posterior ocellus; vestigial anterior ocellus a small dark spot, lappets brown (Figure 16A); antennae brown. Pronotum brown with rugosities, posterior corners rounded (Figure 16A); wing membrane clear, pale brown, with veins brown; legs mostly brown, with joint of femur and tibia, distal tibia and tarsi darker. Abdominal segments brown (Figure 16B).

**Male genitalia.** Tergum 9 with a pair of sparse sensilla patches posteriorly, median broad (Figure 17B). Tergum 10 with few sensilla basiconica posteriorly (Figure 16B,C). Posterior margin of sternum 9 sclerotized, pale brown, fringed with short hairs; hammer oval-shaped, slightly elevated in lateral view, surrounded by short hairs (Figure 16D). Paraproct finger-like, upcurved at middle, strongly sclerotized, with a rounded tip (Figure 16E,F and Figure 17A). Aedeagus mostly membranous, basoventral area with a small patch of hairs; median sclerite absent (Figure 17B,C); Y-arm much longer than Y-stem, approximately 3× as long as Y-stem, with a wide base, curved outward and upward, tapering to apex; lateral sclerites longer than Y-arm, connected with basal Y-arm (Figure 17B–D).

**Female.** Unknown.

**Distribution.** China (Shaoguan City).

**Etymology.** The specific epithet yunjishan derived from the Yunjishan (Guangdong Province, China), the type locality where the holotype was collected. The name is used as a noun in apposition and is gender-neutral.

**Remarks.** The new species resembles the Vietnamese *S. biocellata* [8], which is distributed in Vietnam, shares a similar sensilla basiconica patch and aedeagal sclerites. It can be easily separated from *S. biocellata* by the Y-arms, which are approximately three times as long as the Y-stem in the new species, whereas they are approximately 2.5 times as long as the Y-stem in *S. biocellata*, and by the absence of a slender median sclerite on the ventral surface, which is present in *S. biocellata* (Figure 3 and Figure 4 in Stark & Sivec 2008 and Figure 17B–D).

### 3.4. Identification Key to Chinese Species of Sinacroneuria Based on Adult Male Characters

* Specimens or photographs of *S. complana*, *S. fujianensis*, *S. lateralis*, and *S. yiui* were not available for this study; therefore, the character states used for these species were taken solely from published descriptions and illustrations.

1. Biocellate……………………………………………………………………………………………………………..........2
  Triocellate……………………………………………………………………………………………………....................4
2. T10 with a distinct patch of sensilla basiconica ............................……...……………………......………………….3
  T10 with only a few sensilla basiconica posteriorly (Figure 16A)…........…...……...….........*S. yunjishan* sp. nov.
3. Y-arms of aedeagus longer than lateral sclerites (Figure 3H)………..………………...…...…………….*S. obscura*
  Y-arms of aedeagus shorter than lateral sclerites (Figure 6B)……………………...………..*S. dayaoshan* sp. nov.
4. Paraproct with a stout tip ……………………………………………...…………………...………………................ 5
  Paraproct with a sharp tip ………………………………………………………………...……………….............…. 7
5. Head without a dark spot in ocellar triangle………………………….…………………....……....*S. quadriplagiata*
  Head with a distinct spot in ocellar triangle…………………...……….………………...………...………………..6
6. Y-arms longer than lateral sclerites (Figure 3N)…………………………………………………..…..…….....*S. wui*
  Y-arms shorter than lateral sclerites (Figure 3G)…………………...……………………....……..*S. longwangshana*
7. T9 with only a few sensilla basiconica posteriorly……………………………………………………...……….......8
  T9 with a distinct patch of sensilla basiconica .…………………………………………………...……………….....9
8. Lateral sclerites of aedeagus slender and nearly equal to the Y-arms (Figure 2 in [10])……………*S. complana*
  Lateral sclerites of aedeagus indistinct (Figure 9B)…………........…..……...…………..…*S. fengyanghu* sp. nov.
9. T9 with a pair of sensilla basiconica patches…....………...……...................................…………………………..10
  T9 with a sensilla basiconica patch………………………………………….………….…………………………....11
10. Sensilla basiconica patch on T9 narrowly divided (Figure 2I) ………….………………….….....……*S. orientalis*
  Sensilla basiconica patch on T9 widely divided (Figure 1A in [5]) …………….….…………….......*S. fujianensis*
11. Median sclerite absent…..………………...…………………………………………………………………………...12
  Median sclerite present………………………………………………………..…………………………………........14
12. Paraproct finger-like, slender and pointed (Figure 3B)………………………………………...….......*S. bicornuata*
  Paraproct short and triangular, with sharp, sclerotized tip…………………………………………………….....13
13. Y-stem about one-third the length of the Y-arms (Figure 3F)……………………………………....*S. longiprojecta*
  Y-stem almost as long as the Y-arms (Figure 2F in [5]) …………………...…..……………………….....*S. lateralis*
14. Y-stem almost as long as the Y-arms………………….……………………………………………………………...15
  Y-stem longer than the Y-arms………………………………………...…………………………………..................16
15. T10 with a sensilla basiconica patch completely divided (Figure 2A) ...………………….….....………*S. aequalis*
  T10 with a sensilla basiconica patch divided anteriorly (Figure 2E)………………………………..................*S. lii*
16. Y-arms with apices curved inward ………………..………………………………………………………………....17
  Y-arms with apices curved outward …………………………………………………………………………………19
17. Head light brown, without dark spots (Figure 1J)……………………………………………..................*S. parallela*
  Head light brown, with dark spots ……………………………………………..……………………………………18
18. Y-arms about 4× as long as Y-stem (Figure 3C) ………...………………………………………………*S. dabieshana*
  Y-arms about 2× as long as Y-stem……………………………………………………………………………….*S. yiui*
19. Head with a large stigma from M-line to posterior ocellar triangle (Figure 11B)……*S. magnimaculata* sp. nov.
  Head with a small stigma from M-line to posterior ocellar triangle.…….……………………………………….20
20. Lateral sclerites of the aedeagus longer than Y-arms (Figure 14B) …………………….........*S. similiwui* sp. nov.
  Lateral sclerites of the aedeagus shorter than Y-arms……………………………………………………………...21
21. Aedeagus with a pair of small triangular median sclerites (Figure 3D) ………………………......*S. huzhengkuni*
  Aedeagus with a single median sclerite……………………………………...………………………………………22
22. Median sclerite claviform with a globular base (Figure 3L).……………………………………………......*S. sinica*
  Median sclerite crescent-shaped (Figure 3M) …………………………………………………...………*S. transversa*

## 4. Discussion

Previous taxonomic studies have substantially improved our knowledge of *Sinacroneuria* in China; however, the diversity of the genus remains incompletely documented. Before the present study, *Sinacroneuria* comprised 20 described species worldwide, of which 18 were recorded from China. The discovery of five new species increases these numbers to 25 species worldwide and 23 in China, indicating that the genus remains insufficiently explored, particularly in mountainous regions where collecting has been relatively limited. The present revision also clarifies several taxonomic issues by re-examining historical material, updating species diagnoses, revising remarks for problematic taxa, and providing an identification key covering all currently recognized Chinese species.

Despite the present revision, several taxonomic problems remain unresolved. For a number of historical species, available information is still limited by incomplete original descriptions, the scarcity of reliably identified material, or the loss of type specimens. Additional material, especially from type localities and adjacent regions, is needed to evaluate diagnostic characters, assess intraspecific variation, and further clarify species boundaries within *Sinacroneuria*. Future studies incorporating newly collected specimens and additional morphological data will contribute to a more comprehensive understanding of species diversity and distribution in this genus.

## Data Availability

Specimens are available at the Entomological Museum of China Agricultural University, Beijing, and the Henan Institute of Science and Technology, Xinxiang, China. The Entomological Museum of China Agricultural University, Beijing, China; the Henan University of Science and Technology, Luoyang, Henan, China; the Insect Collection of Henan Institute of Science and Technology, Xinxiang, China; the Hungarian Natural History Museum, Budapest, Hungary; and the National Animal Collection Resource Center, Institute of Zoology, Chinese Academy of Sciences, Beijing, China.
